# Responses to COVID-19 with probabilistic programming

**DOI:** 10.3389/fpubh.2022.953472

**Published:** 2022-11-21

**Authors:** Assem Zhunis, Tung-Duong Mai, Sundong Kim

**Affiliations:** ^1^School of Computing, KAIST, Daejeon, South Korea; ^2^Data Science Group, Institute for Basic Science, Daejeon, South Korea; ^3^Samsung Electronics, Seoul, South Korea; ^4^AI Graduate School, GIST, Gwangju, South Korea

**Keywords:** COVID-19, probabilistic programming, SEIRD model, non-pharmaceutical intervention, simulation, economic impact

## Abstract

The COVID-19 pandemic left its unique mark on the twenty-first century as one of the most significant disasters in history, triggering governments all over the world to respond with a wide range of interventions. However, these restrictions come with a substantial price tag. It is crucial for governments to form anti-virus strategies that balance the trade-off between protecting public health and minimizing the economic cost. This work proposes a probabilistic programming method to quantify the efficiency of major initial non-pharmaceutical interventions. We present a generative simulation model that accounts for the economic and human capital cost of adopting such strategies, and provide an end-to-end pipeline to simulate the virus spread and the incurred loss of various policy combinations. By investigating the national response in 10 countries covering four continents, we found that social distancing coupled with contact tracing is the most successful policy, reducing the virus transmission rate by 96% along with a 98% reduction in economic and human capital loss. Together with experimental results, we open-sourced a framework to test the efficacy of each policy combination.

## 1. Introduction

The ongoing COVID-19 pandemic is one of the most challenging pandemics in human history, infecting more than 170 million people worldwide with more than 3.5 million fatalities as of May 30, 2021 (1). Rapid and easy transmission of COVID-19 leads to a high and fast-growing caseload, overwhelmingly straining the healthcare systems of many countries. Governments are pushed to apply prompt and effective interventions to protect public health. Such policies include lockdown, social distancing, contact tracing, hygiene, and mask mandates. However, countries differ on these measures and their stringency due to differences in public acceptance, the political climate, or government priority. Thus, many interventions were applied considering the individual socioeconomic status of countries. Furthermore, most countries lacked experience in handling the pandemic, only a handful have successfully brought the pandemic under control. The world has witnessed how the initial response to the virus dictated the trajectory of the virus spread.

Apart from its health impact, coronavirus has affected the economic state of the world with various restrictions imposed by governments to mitigate the virus spread. The pandemic already caused a bigger recession than the Great Depression (2). As reported by Mandel et al. (3) lockdown generates more than a 33% drop in global output at its peak and more than a 9% drop in annual GDP. Furthermore, adverse economic effects of lockdown could even diffuse to the neighboring countries by supply chains (4). Thus, governments should carefully take economic context into account when making policy decisions.

This paper proposes a probabilistic programming method to evaluate the strategies imposed by different countries and point out which policies are the most successful in the initial response to the crisis. To provide insights on the effectiveness of initial responses to the pandemic, we analyzed data from 10 countries covering four continents. Moreover, we present a method to balance economic trade-offs of adopting specific policies by providing a generative model that considers the economic context of a given country. Given the recent focus on vaccination efforts, we also examine the effect of vaccination in the containment of the coronavirus in Israel and the United States.

**Figure 1:**
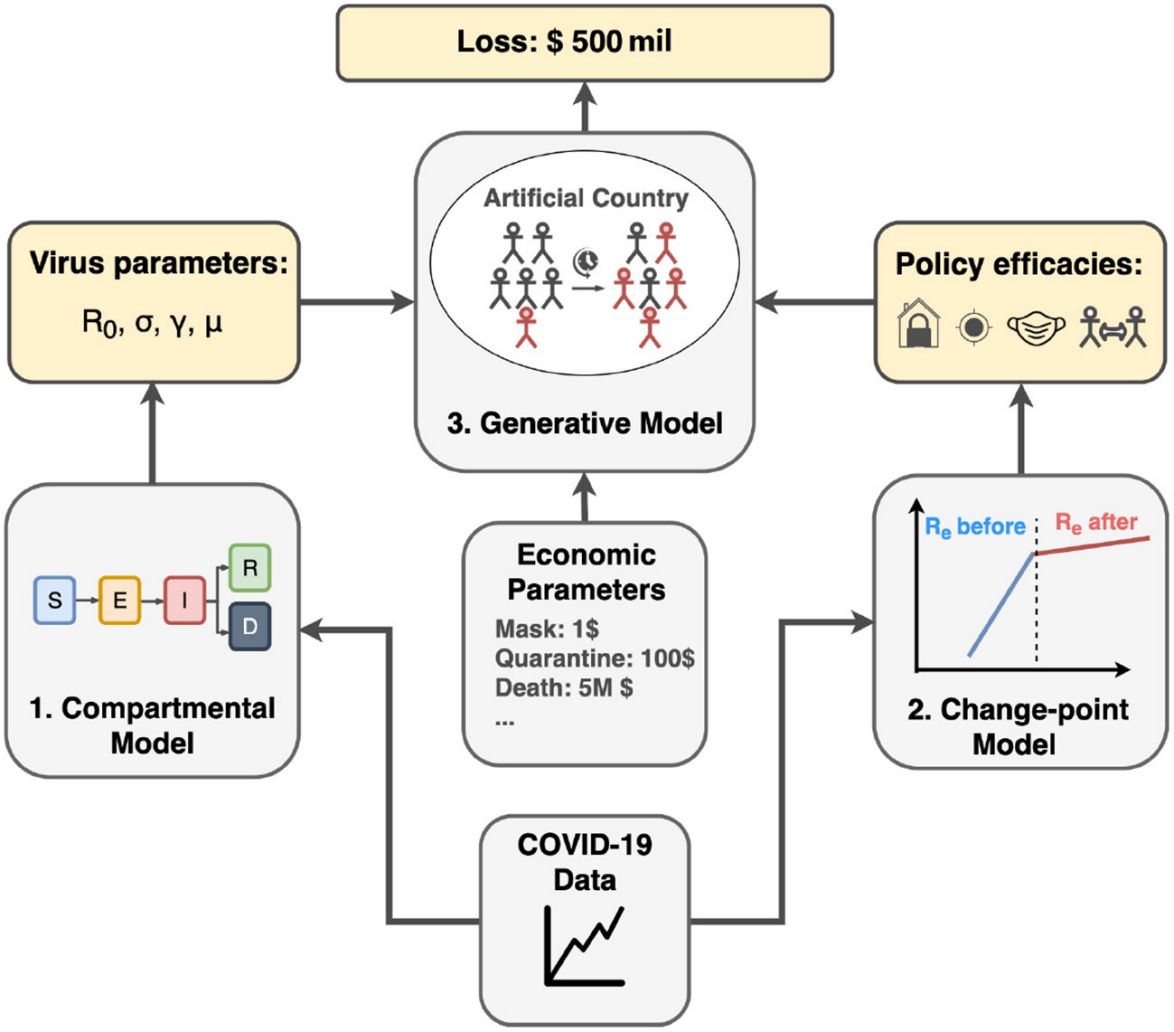
Project pipeline. First, we infer COVID-19 related parameters such as basic reproduction number *R*_0_, incubation rate σ, recovery rate γ, and mortality rate μ using the compartmental model. Second, we apply the change-point model to infer policy efficiencies from different countries. Finally, using inferred parameters from previous steps and economic parameters from real-world data, we run the generative model in artificial country simulation to estimate the economic cost for different policy combinations.

To quantitatively express and analyze the success and failure of different countries, we utilize a probabilistic approach to tackle the COVID-19 transmission dynamics. As illustrated in Figure 1, our approach has three major components:

1. Infer COVID-19 statistics by the compartmental model (Section 4).
2. Estimate policy strengths by the change-point model (Section 5).
3. Simulate virus in the context of policy combinations considering the economic loss by the generative model (Section 6).

The compartmental model is to understand the representative statistics of the virus transmission dynamics, including recovery time, incubation time, reproduction number (*R*_0_), and mortality rate. We infer the baseline statistics by fitting the SEIRD compartmental model on the Swedish data before the Swedish government imposed any policies. We assume that these statistics represent the original virus features unaffected by any human interventions.

With change-point models (5, 6), we estimate the strength of the policies applied to curb the virus spread. Countries around the world impose various interventions with different degrees. Furthermore, the populations worldwide are largely not homogeneous; therefore, the same policy could have different outcomes in different populations. Instead of capturing all these complicated factors, we choose cases where a particular policy can viably represent the upper bound of these policy's efficiency, i.e., the maximum reduction in infection rate. This provides a good idea of how effective each policy would be if applied in full force. To find these upper bounds, we investigate the countries with a successful initial response to the pandemic that stringently applied a given measure, such as China for lockdown or Singapore for social distancing. As these countries curbed the first wave of the spread by firmly applying a particular measure, we can consider the effect in these countries as the maximum effect the measure could perform. The measure will introduce an abrupt change in caseload growth with a significant drop in growth rate, starting from a *change point* in the timeline. We utilize the growth rate before and after this *change-point* to detect the effect of each measure. We run several experiments on countries with different initial responses to the pandemic. The efficiency of the initial policies is represented in terms of the transmission rate change after the policy establishment. Inference result suggests that all major interventions are effective in reducing the virus spread. For example, contact tracing coupled with social distancing yields a 96% reduction in the virus transmission rate, achieving the same effect as lockdown and outperforming all other policies.

The last part of our study includes simulating the virus in an imaginary country that follows all virus and policy statistics inferred from the previous parts. Our generative model is to support decision-makers to solve optimization problems having opposing objectives: public health and the economy. Stringent measures indeed incur economic collapse, but loosening the measures could lead to a devastating crisis. Therefore, the trade-off should be considered carefully. Our model predicts the trajectory of the pandemic, including cases, deaths, and recoveries. Moreover, we incorporate the economic cost into the simulation to address the economic trade-off of policy establishments. By controlling parameters, we estimate how the pandemic plays out in different scenarios and conclude which policy combination can effectively mitigate the virus in public health and economic dimensions. Simulation results suggest contact tracing coupled with social distancing incurs the lowest economic and human capital loss.

With all these analyses, we provide a simple but insightful model to analyze several features of a pandemic: severity of the disease, policy efficiency, and economic impact. This will help to understand the success and failure of each country in its response to the pandemic. It could be used as a playbook to better prepare for a possible pandemic in the future. For reproducibility, the code and datasets used in the paper are available at: https://git.io/JGcPW.

The rest of this paper is organized as follows. In Section 2, we review related work. In Section 3, we introduce the dataset. In Sections 4 and 5, we propose our compartmental model and estimate policy strength by change-point model, respectively. With this model and economic viewpoint, we simulate an artificial country by changing policies in Section 6. We conclude the paper in Section 7.

## 2. Related work

### 2.1. Compartmental models

Most of the epidemic models divide the target population into a certain number of compartments, consisting of individuals with identical statuses concerning a given disease. The foundations of the entire approach to epidemiology based on compartmental models were laid by public health physicians in the early 1900s. One of the first applications of the compartmental model was made by R. Ross, who demonstrated the dynamics of the transmission of malaria between mosquitoes and humans and consequently was awarded the Nobel Prize in Medicine in 1902 (7, 8). Since then, compartmental models are still widely used to simulate the spread of a variety of infections (9).

One of the most popular extensions of the SIR model is the SEIR model (10), a traditional method used to simulate infectious disease that incubates inside the hosts for a while before the hosts become infectious. The SEIR model considers the incubation period by introducing a new compartment *E* (Exposed) to the compartmental system. This model and its modifications were already adapted to simulate the COVID-19 virus in many countries (11–13). In this work, we adopt a widely-used modification of the model—SEIRD (14) with the death compartment *D*. More recently, a SEIRD model with relaxed parameters has also been proposed to consider the rapidly changing social scenario arising from the period of the COVID-19 (15). Likewise, ameliorating compartment models according to the scenario would be a promising direction to study.

### 2.2. Probabilistic algorithms

The Markov chain Monte Carlo (MCMC) is a large class of sampling algorithms widely used for probabilistic problems. MCMC was first introduced in 1953 as a new method to simulate the distribution of states for the system of idealized molecules (16). However, the application of the algorithm did not limit itself to the physics field. It was later adapted and generalized by Hastings (17) to focus on statistical problems, opening its application to a wide range of domains. Due to its ability to handle complex types of analyses, the MCMC approach was widely used in finance (18, 19), communication (20, 21), computational biology (22), linguistics (23, 24), and other fields with probabilistic settings. By no surprise, these methods are widely popular for estimating effects in complex epidemiological analyses as well (25–27). For example, Cauchemez et al. (28) has shown how to model influenza transmission using the Bayesian MCMC approach, and lots of variations of MCMC methods were used to infer features of an Ebola virus and analyze its transmission mechanism (29, 30). Recent reports are also benefited from the Bayesian MCMC methods to infer COVID-19 virus transmission dynamics. Zhou et al. (31) implemented such inference based on a probabilistic compartmental model using daily confirmed COVID-19 cases and applied it to six states of the United States.

MCMC algorithms are also successfully applied to change-point models. The objective is to detect the abrupt property changes lying behind the time-series data (32). Recent work showed that MCMC algorithms with Bayesian parameter inference could be used to detect change-points in COVID-19 spread using SIR and SEIR epidemiological models of South Africa (33). According to their results, South Africa experienced two change-points: the first at the time of the national lockdown and the second after the massive screening and testing program. Dehning et al. (34) adopted a similar approach to the case study of coronavirus spread in Germany by utilizing SIR models with MCMC sampling for detecting change-points in effective growth rate that correlates well with the times of publicly announced interventions. Following these examples, we applied the change point model with an extended SEIRD epidemiology model to identify where policies affected the COVID-19 virus transmission rate in 10 countries with different interventions.

### 2.3. Policy strength estimation

A wide range of work was done to estimate the efficiencies of the policies imposed by different countries to prevent COVID-19. Many of them were focused on the individual country cases considering their unique demographic features (35–37), while other reports compared many countries by the independent effects of a single category of policy (38–40). For instance, Iwata et al. (41) used Bayesian method analysis. They did not reveal the effectiveness of school closures that occurred in Japan in mitigating the risk of coronavirus infection in the nation. Another recent work by Sharov et al. (42) used a modified SIR model to compare the effectiveness of lockdown measures introduced during the coronavirus pandemic in 13 European countries, comparing them to two baseline countries (Sweden and Iceland) that did not implement the lockdown policies. For evaluation, this work used the herd immunity level and time of formation to indicate the effectiveness of lockdown measures (42). According to Sharov's results, lockdown and no-lockdown modes of containment led to roughly similar results.

There are also reports considering multiple policies across the globe (43, 44). One example is work by Flaxman et al. (45), which investigates effects of applied non-pharmaceutical interventions (NPIs) across 11 European countries for the period from the start of the COVID-19 epidemics. According to their results, major non-pharmaceutical interventions, like lockdowns, have had a significant effect on reducing the transmission of the virus. However, a subsequent study by Haug et al. (46), which assessed the efficacy of 6,068 NPIs across 226 countries and gave a detailed analysis of the country-specific “what-if” scenarios, showed different results. They analyzed the impact of government interventions on the effective reproduction number *R*_*t*_ by combining several analytical approaches. By utilizing statistical, inference, and artificial intelligence tools, they concluded that combinations of some less disruptive and less costly NPIs could be as effective as more expensive and harsh ones like national lockdowns. Brauner et al. (47) came to the same conclusion by analyzing 41 countries during the first wave of the pandemic. According to their study, less harsh NPIs can be more effective in mitigating COVID-19 transmission than more strict stay-at-home orders (47). Singh et al. (48) exploited the spatial and temporal variation in the introduction and lifting of non-pharmaceutical interventions (NPIs) across counties using a staggered difference-in-differences (DID) approach. They compared US counties with NPIs in place (treated) with counties that do not have NPIs in place (control) before and after implementation. Enabled by datasets with rich population characteristics, they stratified the datasets into several groups and analyzed the impact of implementing and lifting NPIs by the population groups they target. However, as we will discuss in Section 5, it takes a certain amount of lagging time to see the effects of NPI implementations. More meaningful analysis can be obtained with DID by considering the delay.

However, one of the significant limitations of recent studies is that none of them perform a comprehensive analysis considering the economic factors that affect the efficiencies of the policies. There were some reports regarding the economic cost of the pandemic situation across the globe (49). For example, McKibbin and Fernando (50) simulated a global economic model to explore seven scenarios, which differ in the proportion of the population who become infected or dead. According to their estimations, in a scenario where COVID-19 develops into a global pandemic, the cost of lost economic output begins to escalate into trillions of dollars (51). However, they do not include the effect of policy interventions in their simulations. To address this limitation, we propose another method of cost estimations by involving policy effects in Section 6.

## 3. Dataset

**Figure 2:**
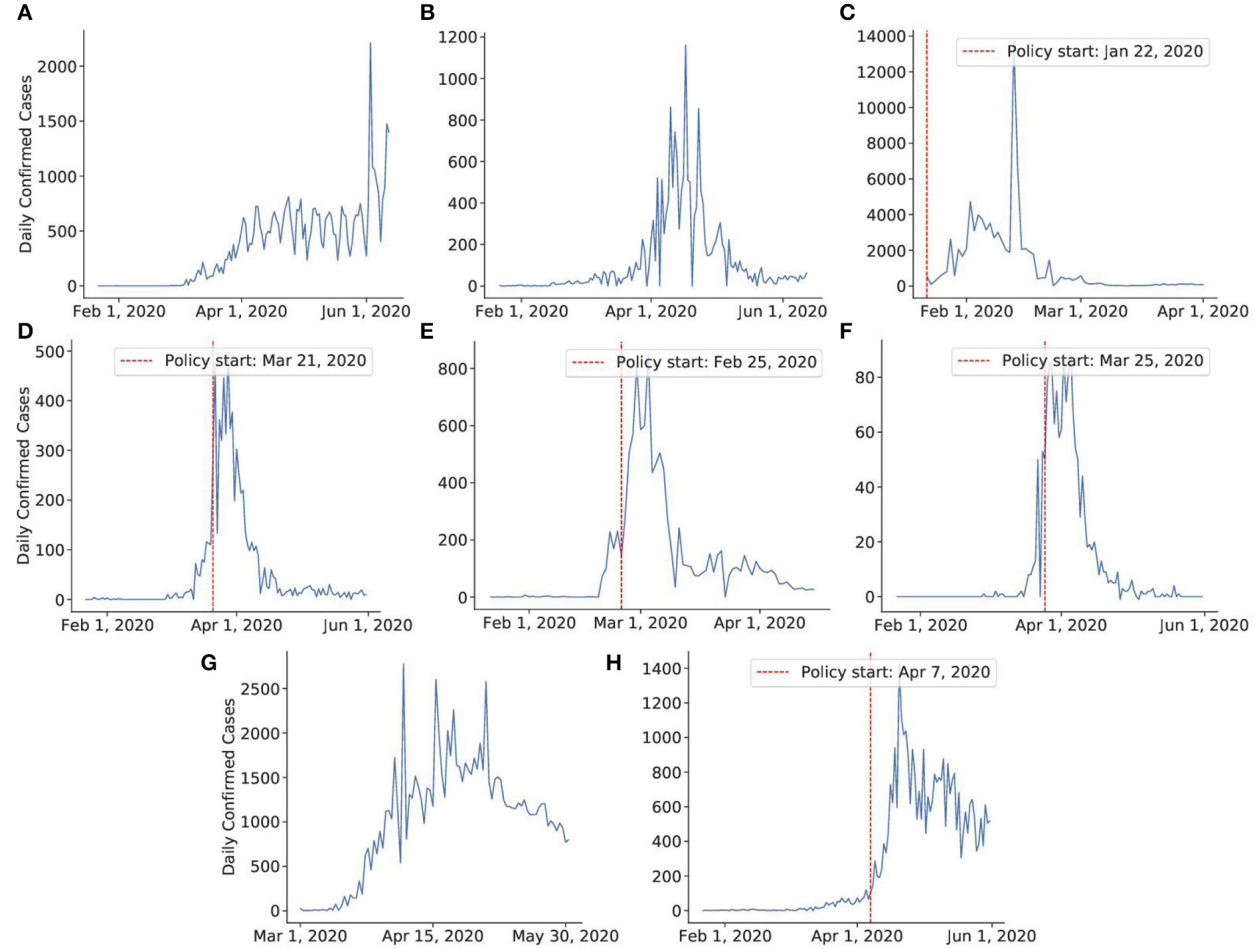
Daily number of confirmed cases of eight countries. Vertical red lines indicate the date when a certain policy was established. In the case of Sweden and Japan, we consider the time period before the policy establishment. For Canada, the policy was established gradually. Thus, the exact dates of policy start for this country are not determined. **(A)** Sweden, **(B)** Japan, **(C)** China, **(D)** Australia, **(E)** Korea, **(F)** New Zealand, **(G)** Canada, **(H)** Singapore.

**Figure 3:**
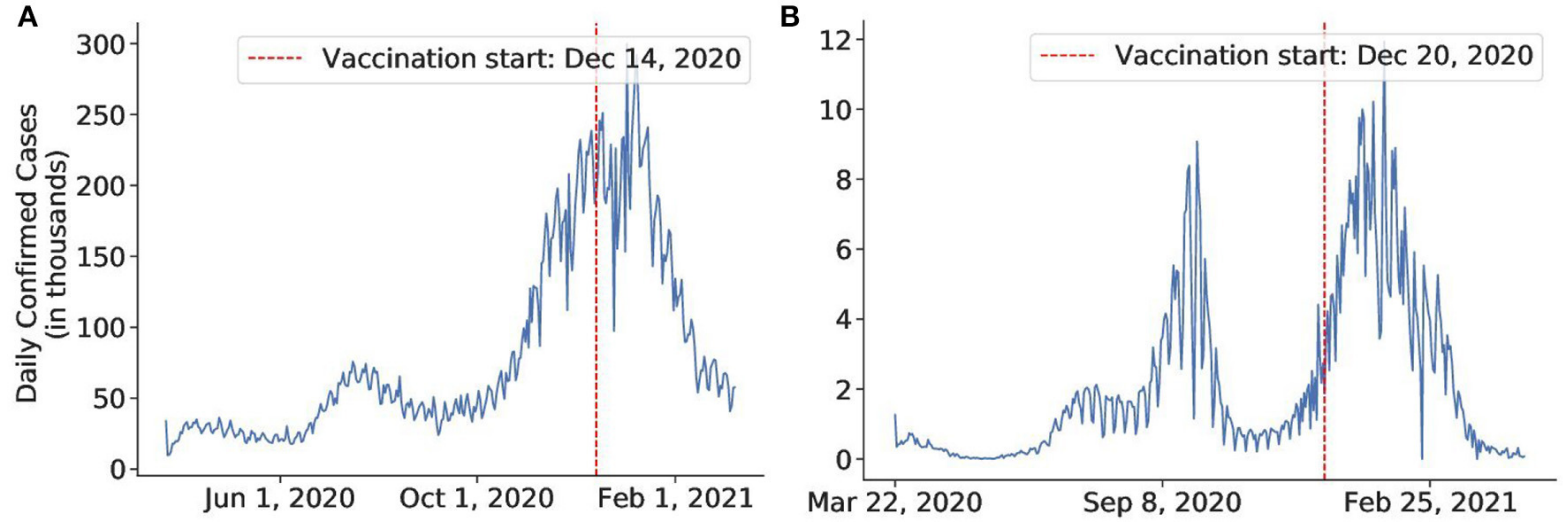
The daily number of confirmed cases of the countries in a timeline in which we investigate the effect of vaccines. **(A)** United States, **(B)** Israel.

For our analysis, we used virus data from two sources that are available online. The first dataset is taken from Kaggle. This COVID-19-related dataset^1^ was collected from the John Hopkins University dashboard^2^ and Worldometers website^3^. The data for Israel and the US was taken from the COVID-19 Data Repository by the Center for Systems Science and Engineering (CSSE) at Johns Hopkins University^4^. Both datasets report the number of confirmed, death, and recovered cases for each day since the first confirmed case across the globe, divided by countries, regions, and provinces. The plots of daily confirmed cases for 10 countries used for analysis can be found in Figures 2, 3.

## 4. Analyze COVID-19 statistics by compartmental model

In this section, we will introduce the proposed methodology to infer SARS-CoV-2 virus statistics. We will first present the SEIRD epidemiological compartmental model and the corresponding probabilistic programming model to infer several virus statistics. Then, we perform inference on the Sweden data to infer the important virus parameters^5^.

### 4.1. Compartmental SEIRD model

The most basic compartmental model, namely the SIR model, uses three compartments of Susceptible (S), Infectious (I), and Recovered (R). Each individual can move from a compartment to another compartment, resembling the progress of the disease. We could use S, I, and R to denote the number of individuals in their respective compartments. For the COVID-19 case, there is an incubation period in which people are infected but not yet infectious. Hence, we adapted the epidemiological SEIRD model (14) for our simulations, extending the SIR model with the E compartment of exposed individuals and the D compartment for deaths.

- Susceptible (S): Individuals in the compartment are neither infected nor immune to the diseases and, hence, could contract the disease. If the susceptible individuals contract the disease (*via* contact with an infectious individual), they progress to the Exposed compartment.
- Exposed (E): Individuals in the compartment are infected but unable to pass the disease to susceptible individuals. If the Exposed individuals finish their incubation period and can infect others, they progress to the Infectious compartment.
- Infected (I): Individuals in the compartment are infected and pass the disease to susceptible individuals. If the Infectious individuals recover from the disease and carry immunity or die from the disease, they progress to the Recovered (or Resistant) and Dead compartments, respectively.
- Recovered (R): Individuals in the compartment are immune to the disease. If the Recovered individuals lose their immunity, they progress to the Susceptible compartment.
- Dead (D): Individuals in the compartment cannot progress to any other compartment.

The below differential equations (Equations 1–5) describe the transition between compartments (14).


(1) $$\begin{array}{ll} \frac{dS}{dt} & =-\frac{R_{e}\gamma S}{N}I+\alpha R, \end{array}$$

(2) $$\begin{array}{ll} \frac{dE}{dt} & =\frac{R_{e}\gamma S}{N}I-\sigma E, \end{array}$$

(3) $$\begin{array}{ll} \frac{dI}{dt} & =\sigma E-\gamma I, \end{array}$$

(4) $$\begin{array}{ll} \frac{dR}{dt} & =\gamma \left(1-\mu \right)I-\alpha R, \end{array}$$

(5) $$\begin{array}{ll} \frac{dD}{dt} & =\gamma \mu I, \end{array}$$

(6) $$\begin{array}{ll} N & =S+E+I+R+D, \end{array}$$

where the effective reproduction number (*R*_*e*_) is the expected number of people that each infected individual can transmit the virus to during the outbreak, the basic reproduction number (*R*_0_) is the natural reproduction number when there is no intervention, the incubation time ($t_{E}=\frac{1}{\sigma }$) is the average time in which an individual is exposed but not yet infectious, the recovery time ($t_{I}=\frac{1}{\gamma }$) is the average time after which an the infected case become concluded (recovered/dead), the case fatality proportion μ is the proportion of fatal cases among all concluded cases, and the waning time ($t_{R}=\frac{1}{\alpha }$) is the time that recovered individuals retain immunity. Since we deal with the initial stages of the pandemic, we assume people carry immunity to the disease upon recovery (α = 0) and the population stays constant over time and is equal to N.

Substantial amounts of COVID-19 cases are not reported due to testing availability and testing strategy. Hence, the exact total number of COVID-19 cases is unknown and typically not uniquely determined from the number of confirmed cases (52–54). Korolev (53) emphasizes that neglecting unreported cases leads to biased parameter estimation, so it is important for our model to address this issue.

To distinguish observable cases among all possible cases, we use a parameter called response rate ρ, which is the probability of a case being reported. At each timestamp (in our case, each day), if the SEIRD model estimates the number of new cases (transition from S to E) to be S2E and the number of recovered cases (transition from I to R) to be I2R, our model, the number of reported confirmed cases and recovered cases will be corrected by the response rate:


(7) $$\begin{array}{l} \text{Newly reported confirmed cases}\sim Binomial\left(S2E,\rho \right) \end{array}$$

(8) $$\begin{array}{l} \text{Newly reported recovered cases}\sim Binomial\left(I2R,\rho \right), \end{array}$$

where *Binomial*(*n, p*) is the discrete probability distribution of the number of successes in a sequence of n experiments. Here, a reported case can be understood as a success, and each case can be understood as an experiment. Response rate ρ is inferred together as a parameter for our SEIRD model. Since it varies widely across countries, the estimated value is used internally within the country and is not generalized for others.

### 4.2. Scaling up with probabilistic programming

To implement the probabilistic models, we used the probabilistic programming language Pyro (55). For this particular inference task, we adopted Pyro's Epidemiology framework (56) for scaling up our experiments with a restricted class of stochastic discrete-time discrete-count compartmental models. This framework uses the Markov Chain Monte Carlo (MCMC) algorithm to fit the SEIRD model to infer COVID-19-related parameters: reproduction number *R*_0_, recovery time, incubation time, transmission rate, and mortality rate.

#### 4.2.1. Summary of the method

MCMC is a stochastic algorithm that repeatedly generates random samples describing the distribution of parameters of interest (in our case, COVID-19 related parameters), where a new sample is generated based on the previous sample, thereby creating a Markov chain. The Markov chain has a stationary probability *p*_*S*_(*x*) such that if the chain ever arrives at *p*_*S*_(*x*), it will keep sampling from *p*_*S*_(*x*) forever. Therefore, the goal of MCMC is to design a transition probability to make the stationary distribution equate the target probability [i.e., *p*_*S*_(*x*) = *p*(*x*)]. Starting from an initial random sample, the algorithm guides the Markov chain to the stationary distribution, which we force to be the same as the target distribution (57).

A popular instance of the MCMC method is the Metropolis-Hastings algorithm that uses sampled proposal probability distribution (also called the kernel), followed by an acceptance criterion that chooses to accept or discard the new sample by comparing how likely the proposal distribution is to differ from the true next-state probability distribution. This criterion is implemented by an *acceptance ratio*, the probability for which we accept the new sample. If the proposal distribution is closer to the true distribution, we set a higher ratio to accept the new sample. For optimizing the sampling process, we used an instance of the Metropolis-Hastings algorithm, namely the Hamiltonian Monte Carlo (HMC) algorithm with the No-U-Turn Sampler (NUTS). The HMC algorithm avoids random walk behavior by taking steps informed by the first-order gradient information (58). It utilizes an approximate Hamiltonian dynamics simulation, which is then corrected by a Metropolis acceptance step (59, 60). HMC reduces the correlation between successive sampled states, allowing the algorithm to converge much faster with fewer Markov chain samples. However, since HMC is highly sensitive to two hyper-parameters: step size and the number of steps, the No-U-Turn Sampler (NUTS) is used to adaptively set these parameters (58). Thus, we can perform HMC without any manual hyperparameter tuning.

#### 4.2.2. Algorithmic presentation

From the observed COVID-19 trajectory (number of cumulative confirmed cases, recovered cases, fatalities over time), we apply HMC-NUTS to infer the right parameters to describe the development of the pandemic. Considering the pandemic follows the SEIRD model, Algorithm 1 describes how the HMC-NUTS algorithm estimates the model parameters. Here, **y** = [*y*_1_, *y*_2_, …, *y*_*T*_] is a pandemic trajectory with its statistics *y*_*t*_ at time *t*.

**Algorithm 1:**
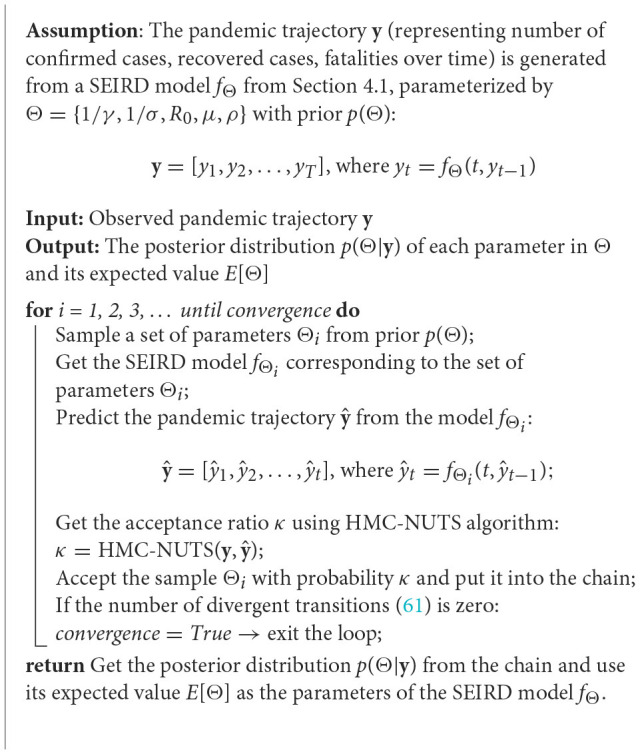
Estimating parameters of the SEIRD model using HMC-NUTS algorithm.

### 4.3. Fitting SEIRD model to Sweden—The reference country

We ran the model to estimate the parameters of the Swedish data before April 1st, 2020. We chose this early stage of the COVID-19 pandemic since Sweden did not impose any strict policies and aimed to achieve herd immunity (62). We assumed that the virus transmission rate was unaffected by any interventions, so we used Sweden as a baseline case and perform the experiments to infer unaffected COVID-19 parameters. To run our probabilistic model, we set the prior according to the estimations of the World Health Organization (63). It was reported that mild cases typically recovered within two weeks, the incubation period was on average 5–6 days, and *R*_0_ was typically around 2. Mortality and recovery rates differed depending on the region and stage of the virus spread, but the case-fatality rate was roughly 2.5%. Prior of the response rate ρ is given as Beta(10, 10), which favors the initial value around 0.5, then converges in the range of 0 to 1.

The obtained posterior virus-related statistics for Swedish data are shown in Table 1. For more accurate results, we ran the model six times and reported the averaged values. The results are reasonable enough to use in our further simulations.

**Table 1:** Estimation results of COVID-19 virus parameters without any interventions.

**Parameter**	**Abbreviation**	**Value**
Recovery time	1/γ	16.33 days
Incubation time	1/σ	5.27 days
Basic reproduction number	*R*_0_	2.64
Case-fatality rate	μ	2.5%

According to model's estimations it takes around 16 days to recover after being infected, 5 days for symptoms to develop after the exposure and mortality rate is equal to 2.5%.

## 5. Estimation of policy strength by the change-point model

Change points in time series denote abrupt variations, and such changes represent transitions that occur between states (5). Change-point detection concerns whether or not a change has occurred or identifying the time of any such change. It is useful in modeling and predicting time series in diverse applications such as human activity analysis, speech and image analysis, medical monitoring, and anomaly detection (6).

This section introduces a change-point detection methodology to quantify the efficiency of the major interventions applied worldwide to mitigate the COVID-19 spread. First, we briefly introduce the concept of estimating the policy strength by referring to the compartmental model and its formula. Second, we describe a probabilistic programming model that detects change-points in the course of the caseload after the country applied NPIs. In the process of detecting change points, a probabilistic programming model can estimate the policy strength together. Next, we elaborate on several countries' data to conclude the efficiencies of investigated policies and give a summary of our findings in the final subsection.

### 5.1. Concept: Policy strength and the change-point

There are several works that explain and compare the transmission of viruses under various situations by using the slope of the log-transformed instances. Both the rate of change of the log-transformed case incidence and the instantaneous reproduction number, *R*_*e*_, are considered important for the investigation of the virus (64). For example, Caspi et al. (65) modeled replication rate (RR) as the slope of the logarithmic curve of confirmed cases to compare the coronavirus spread in different climates. Another study by Gebski et al. (66) observed changes in the slopes of log-transformed incidents of *Staphylococcus aureus* (MRSA) infections in hospitals to evaluate the success of interventions. In this work, we adopt similar strategy by evaluating effectiveness of the interventions by comparing the slopes of the confirmed cases before and after the policy establishment.

We will first explain how to measure the policy strength by referring to the SEIRD compartmental model (see Section 4.1) with some simple assumptions and new terms we describe below. The transmission rate β is the number of susceptible individuals that an infected individual can infect in a day, which is calculated as β = *R*_*e*_γ. At the very beginning, almost everyone in our setting is in the Susceptible compartment, so we can assume that *S* is equal to the total population size *N*, or *S* = *N*. With this approximation, Equation (1) can be rewritten as follows:


(9) $$\begin{array}{ll} \frac{dS}{dt} & =-R_{e}\gamma I=-\beta I \end{array}$$

Additionally, since the incubation period is much shorter than the recovery time, we can ignore the E, R, and D compartments at the initial stage of the simulation. Thus, we can approximate the total population size as *N* = *S*+*I* and Equation (3) becomes:


(10) $$\begin{array}{ll} \frac{dI}{dt} & =\beta I \end{array}$$

The size of the Infected compartment rises exponentially with the rate *w* = β (that is, on day *t*, the number of infected cases is calculated as *e*^β*t*^). Due to the exponential nature, it is appropriate to investigate the case counts using a log scale. In the log scale, the exponential spread is represented as a linear line, with the transmission rate of the virus β represented by the slope *w*.

**Figure 4:**
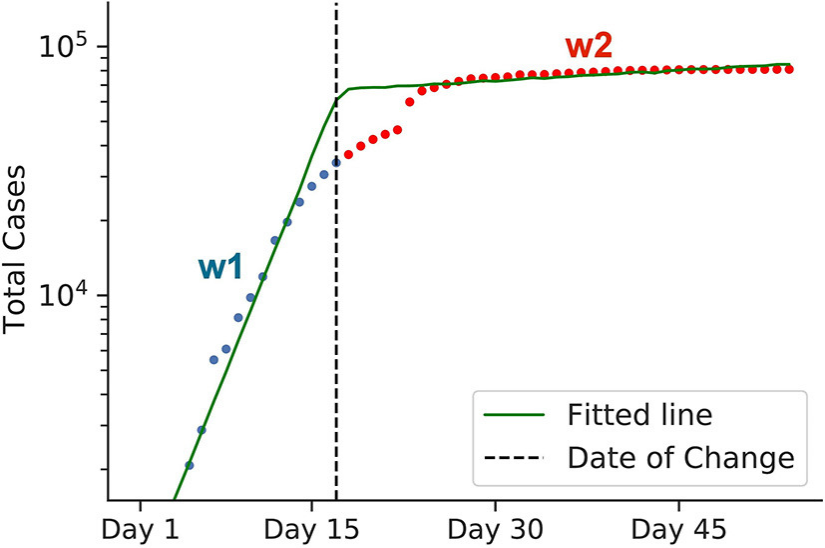
Graphical representation of estimating policy strength. The green line represents a log-scaled cumulative case counts. Due to some interventions, the graph bends at the change-point, as indicated by the black dotted line. The change-point divides the graph into two intervals: blue and red. Both lines have different slopes, *w*_1_ and *w*_2_, respectively, which represent the virus transmission rates before and after the intervention came into effect. To calculate the efficiency of the intervention, we use the formula 1−*w*_2_/*w*_1_. The data for early Chinese cases was used for this example. Here, the calculated 1−*w*_2_/*w*_1_ value represents the efficiency of the lockdown policy of China back in early 2020.

Consider an example in Figure 4. In the beginning, the graph is a steep line with the slope *w*_1_ (representing a rapid, exponential spread). After a corresponding policy is applied, the graph bends and becomes less steep with the slope *w*_2_≪*w*_1_ (slower spread). Therefore, the graph roughly consists of two lines of different slopes, *w*_1_ and *w*_2_, with a separation point in-between, which we call a *change-point* (the black dotted vertical line in the graph). The slope *w*_1_ and *w*_2_ represent the transmission rates before and after the change-point. Since *w*_1_ and *w*_2_ represent the transmission rates before and after the policy takes its effect, we can define the strength of the target intervention in terms of their ratio:


(11) $$\begin{array}{l} \text{Policy efficiency}=1-\frac{\beta_{2}}{\beta_{1}}=1-\frac{w_{2}}{w_{1}} \end{array}$$

Given the incubation period, we expect the policy will show effect after around 2–4 weeks after the policy establishment. In the next subsection, we will introduce a probabilistic programming approach to find the change-point when the policy takes effect.

### 5.2. Implementation: Change-point detection with policy strength estimation

As was mentioned in the related work (see Section 2.2), probabilistic models were successfully used to detect change-points in transmission rates of coronavirus. In the present subsection, we describe the probabilistic programming approach to detect change-points as well as estimating policy strength. Algorithm 2 sketches how the change-point detection model based on the probabilistic programming operates. From the case trajectory of the COVID-19 pandemic, we used the HMC algorithm with NUTS (58) to estimate the change-point τ and slopes *w*_1_ and *w*_2_.

**Algorithm 2:**
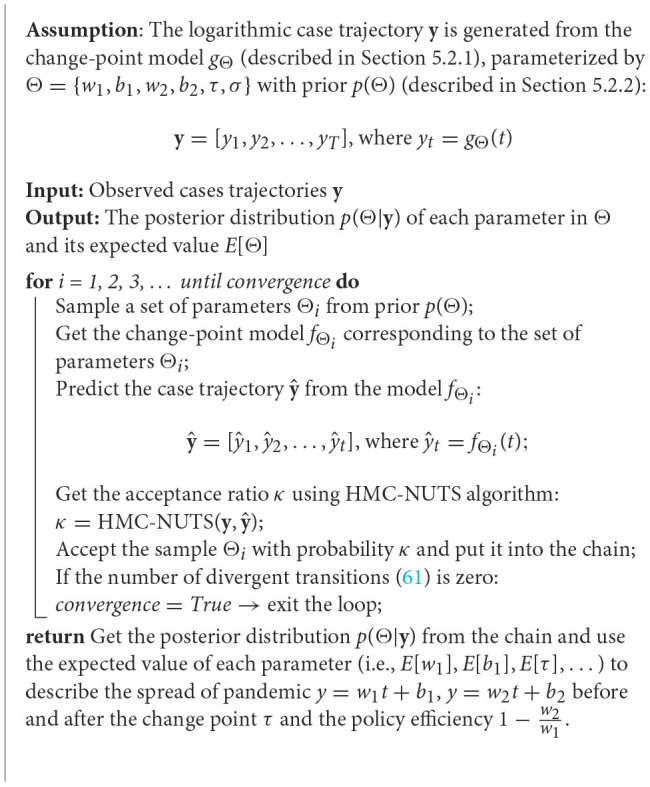
Change-point detection using HMC-NUTS algorithm.

#### 5.2.1. Likelihood choice

In our probabilistic setting, the likelihood corresponds to the log-scaled line of accumulated confirmed cases. We chose piece-wise linear regression and added the StudentT noise, which is more robust w.r.t the outliers than conventional Gaussian noise (67). We define τ as the change-point in the range [0, 1], with 0 and 1 being the start and end of the simulation time period, respectively. The likelihood can be modeled as follows:


(12) $$\begin{array}{l} y=wt+b+\epsilon , \end{array}$$

where


$$\begin{array}{l} w,b=\{\begin{array}{ll} w_{1},b_{1}, & \text{if }t<\tau \\ w_{2},b_{2}, & \text{if }t>\tau \end{array}\\ \epsilon \sim StudentT(2,0,\sigma^{2}). \end{array}$$

Note that the weights *w*_1_ and *w*_2_ correspond to slopes before and after the change-point in Equation (11) and Figure 4. To sum up, the change-point model is parameterized by six factors: *w*_1_, *b*_1_, *w*_2_, *b*_2_, τ, and σ.

#### 5.2.2. Prior choice

Here we illustrate the choice of parameters' priors used as input for our probabilistic model to draw samples from. For weights, we use the normal distribution, with *w*_2_ having the mean equal to zero as we expect the slope to drop significantly after the change-point.


(13) $$\begin{array}{l} w_{1}\sim N(0.5,0.25)\\ w_{2}\sim N(0,0.25) \end{array}$$

For bias terms, we set the priors to be from normal distributions. However, this time, we adjust the bias priors for each country adaptively since bias is sensitive to each country's course of the caseload. We assign the mean of y in the first and fourth quartiles to *m*_1_ and *m*_2_, respectively. For *b*_1_ to be relatively flat the standard deviation *s*_1_ is set to 1 and *s*_2_ is set to 0.25*m*_2_.


(14) $$\begin{array}{l} \begin{array}{c} b_{1}\sim N\left(m_{1},s_{1}\right)\\ b_{2}\sim N\left(m_{2},s_{2}\right) \end{array} \end{array}$$

We use Beta distribution as a prior for the change-point τ and assume that the change is more likely to occur in the second half of the date range. We choose the parameter of the Beta distribution so that the peak of Beta(4, 3) falls to the 60th percentile of the date range.


(15) $$\begin{array}{l} \begin{array}{c} \tau \sim \text{Beta}\left(4,3\right) \end{array} \end{array}$$

The magnitude of the noise is quantified by the standard deviation σ. We put a simple uniform prior for σ.


(16) $$\begin{array}{l} \begin{array}{c} \sigma \sim \text{Uniform}\left(0,3\right) \end{array} \end{array}$$

Using the prior defined above and the actual case trajectory, we can finally estimate the parameters to measure the policy efficiency with the change point (see Algorithm 2).

### 5.3. Inferring maximum efficiencies of major policies with the change-point method

We investigate major initial interventions applied by several countries to mitigate the virus spread. For more accurate results, we chose nine countries presented in Table 2 that strongly imposed corresponding policies, assuming that they were applied to the fullest extend. By investigating the countries that applied a policy most stringently, we find a meaningful upper bound for each policy's efficacy. This upper bound is helpful for policymakers to determine the most appropriate intensity of the policy (more details in Section 6). In this experiment, we focused on five main policy categories:

- *Lockdown*: A lockdown is an intervention that forces people to stay where they are. It includes a gathering ban, closure of non-critical services, and strict transportation restrictions. People cannot freely enter or exit their designated areas, and economic activities are essentially suspended.
- *Social distancing*: Social distancing includes interventions or measures intended to maintain a physical distance between people, including a gathering limit or closure of non-essential services. It can be considered as a partial or a soft lockdown.
- *Contact tracing and social distancing*: Contact tracing is the policy that investigates the close contacts of infected cases and then tests and quarantines them. Investigating the countries with successful contact tracing campaigns revealed that they coupled the contact tracing intervention with social distancing (e.g., South Korea, Australia, and Vietnam). For this reason, instead of addressing contact tracing separately, we merged it with social distancing to be closer to the real-world scenario.
- *Mask and hygiene mandate*: Almost every country imposed a mask mandate sometime in their COVID-19 timeline response. Since it is always coupled with other restrictions, separating the effect of mask mandate from other interventions is a challenging task. Because the change-point method cannot be applied in this case, we proposed a different approach to this issue, discussed in Section 5.4.4.
- *Vaccine*: With the recent roll-out of vaccines worldwide, we also examine the effect of recent vaccination campaigns in Israel and the US. The effectiveness of the vaccines depends on several factors, including the time it takes to approve, manufacture, and deliver them to the population, as well as improvements, and the development of other vaccine variations, and the proportion of the population vaccinated. While there are many reports on the effectiveness of several vaccines in laboratory settings (68, 69), the early effects of the vaccinations on a large scale have yet to be studied in more detail. Assuming that vaccination campaigns can result in the same effects on virus mitigation as any other policy that the government may enact, we also analyze the effectiveness of the vaccination programs by applying the same change-point model.

**Table 2:** Countries and policies used for analysis.

**Policy**	**Country**	**Started from**	**Strength**	**Take effect**
Lockdown	China	Jan 2020	0.98	16 days
Lockdown	New Zealand	Mar 2020	0.95	8 days
Contact tracing and distancing	South Korea	Feb 2020	0.96	8 days
Contact tracing and distancing	Australia	Mar 2020	0.96	10 days
Social distancing/soft lockdown	Canada	Mar 2020	0.70	–
Social distancing/soft lockdown	Singapore	Mar 2020	0.78	20 days
Mask and hygiene	Japan	Feb 2020	0.30	–
Vaccine	US	Dec 2020	0.73	35 days
Vaccine	Israel	Dec 2020	0.88	59 days
No intervention (Herd immunity)	Sweden	Mar 2020	0	–

Policy strength and time to take effect are inferred by change-point model. Countries and their major policies are listed according to the starting time and strength. In extreme policies such as lockdowns, the effect comes quickly, but there are side effects to be described later in Section 6.3.

**Figure 5:**
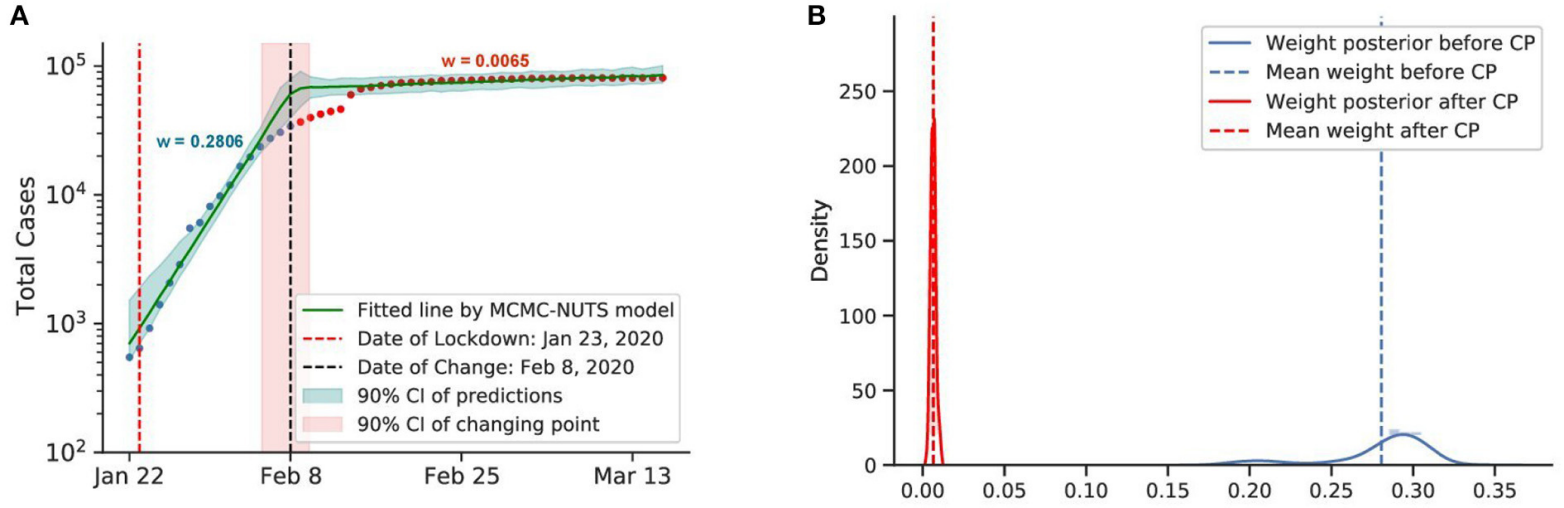
Posterior for China. As the initial epicenter of the outbreak, cases in China skyrocketed in January 2020. China started applying a swift and stringent lockdown from January 23, 2020 (70), starting with the city of Wuhan, and managed to largely bring the outbreak under control in February, 2020. The growth rate was suppressed staggeringly from 0.2806 to a mere 0.0065, with the change-point estimated around February 8, 2020. **(A)** Fitted graph for China with change-point on February 8, 2020. **(B)** Posterior distribution of weights (transmission rate) before and after change-point.

**Figure 6:**
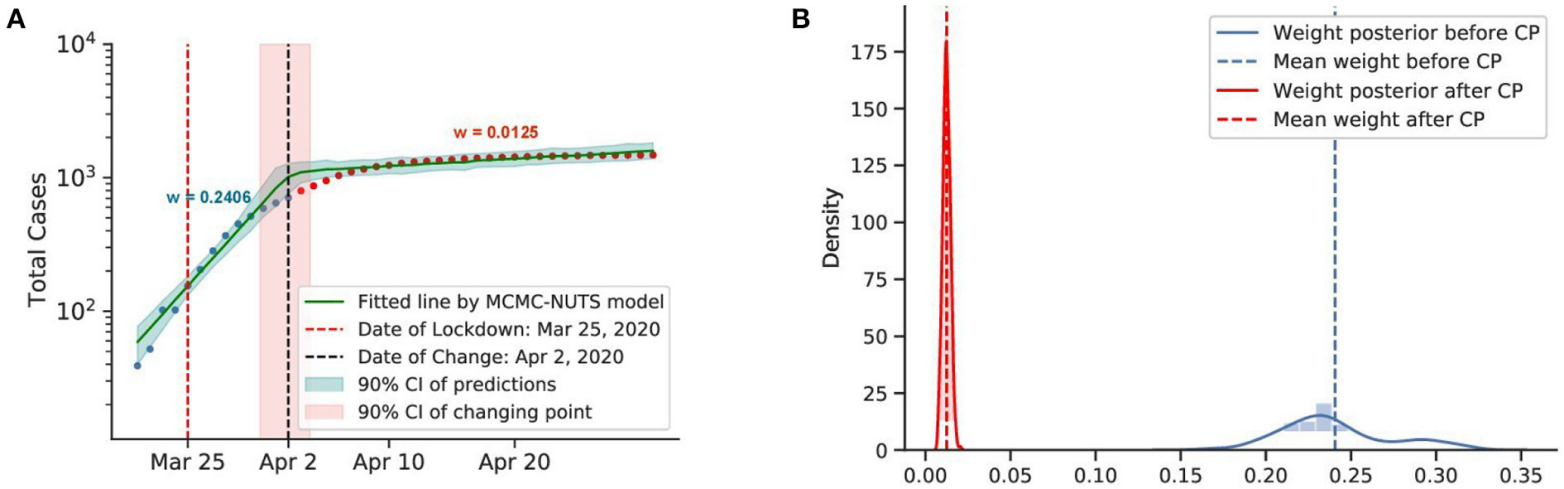
Posterior for New Zealand. After confirming the first case, New Zealand experienced a rapid spread of the diseases. The government imposed a strong lockdown from March 25, 2020 (71) and brought the outbreak under control in February, 2020. The growth rate declined from 0.2406 to 0.0125. The change-point was around April 2, 2020. **(A)** Fitted graph for New Zealand with change-point on April 2, 2020. **(B)** Posterior distribution of weights (transmission rate) before and after change-point.

**Figure 7:**
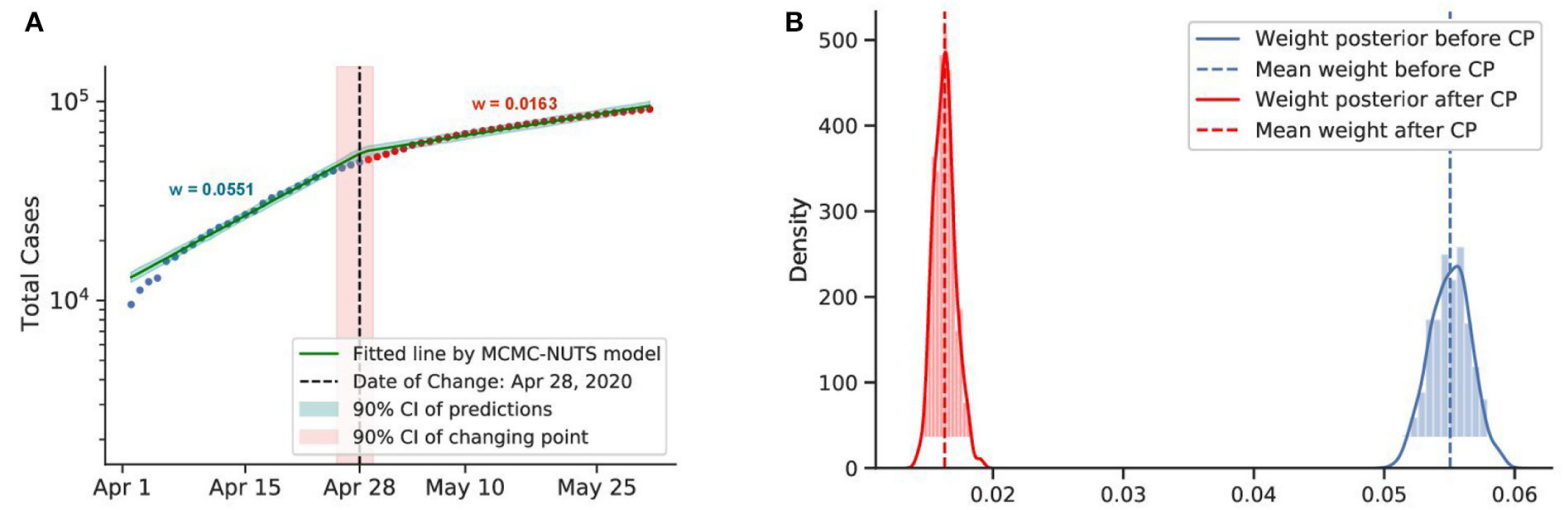
Posterior for Canada. Canada recorded a total of more than 10,000 cases up to early April. The government imposed various restrictions in March-April (72) and reduced the transmission rate from 0.0551 to 0.0163. The change-point point was around April 28, 2020. **(A)** Fitted graph for Canada with change-point on April 28, 2020. **(B)** Posterior distribution of weights (transmission rate) before and after change-point.

**Figure 8:**
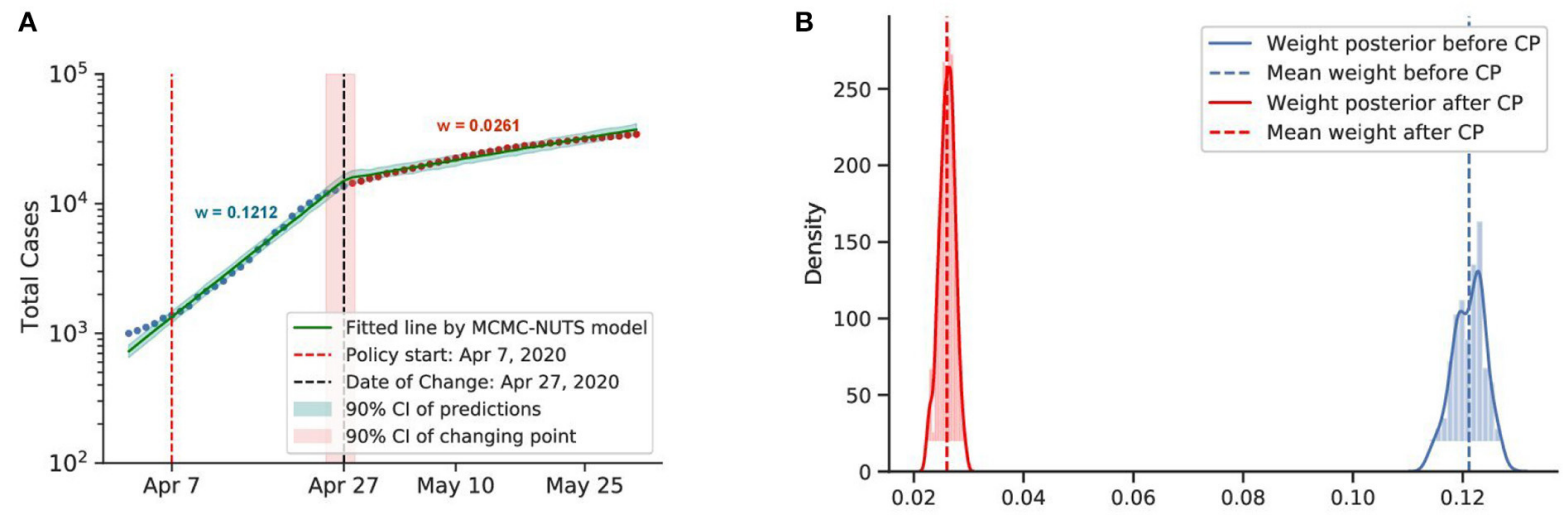
Posterior for Singapore. Singapore saw a rapid growth of the caseload among migrant workers around April 2020, and a “circuit-breaker” was imposed on April 7 (73) that brought the outbreak under control in late April, with the growth rate declining from 0.1212 to 0.0261. The change-point point was around April 27, 2020. **(A)** Fitted graph for Singapore with change-point on April 27, 2020. **(B)** Posterior distribution of weights (transmission rate) before and after change-point.

**Figure 9:**
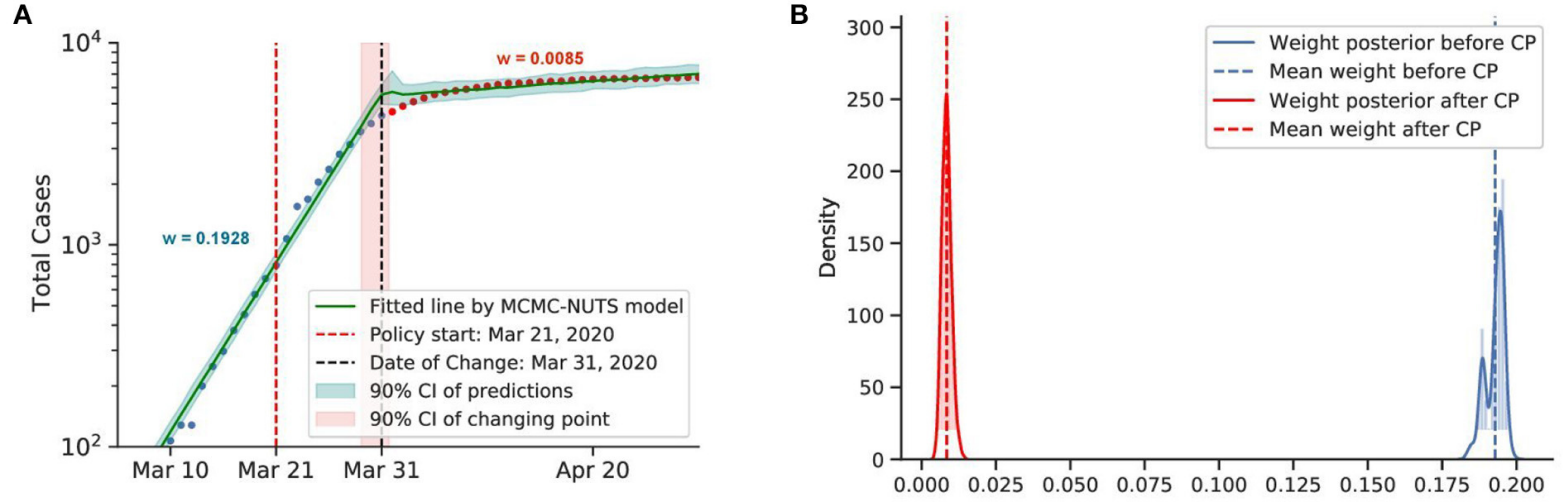
Posterior for Australia. After confirming the first case, Australia recorded almost 10,000 cases before the end of March. With social distancing and contact tracing efforts from March 21, 2020 (74), Australia contained the spread, with a decline in the growth rate from 0.1928 to 0.0086. The change-point point was around March 31, 2020. **(A)** Fitted graph for Australia with change-point on March 31. **(B)** Posterior distribution of weights (transmission rate) before and after change-point.

**Figure 10:**
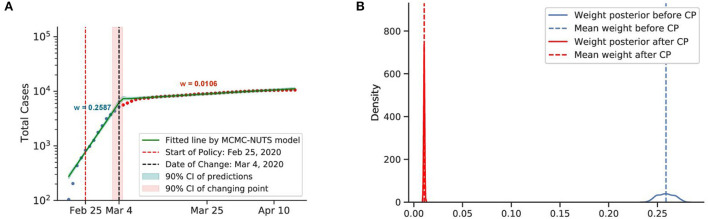
Posterior for South Korea. South Korea was the second epicenter of the outbreak after the super-spreader event of Patient 31. The government relied on social distancing and extensive contact tracing to avoid a stringent lockdown, starting shortly after the Patient 31 event (around February 25, 2020) (75, 76), and brought the outbreak under control in March, with a decline in the growth rate from 0.2587 to 0.0106. The change-point point was around March 4, 2020. **(A)** Fitted graph for South Korea with change-point on March 4, 2020. **(B)** Posterior distribution of weights (transmission rate) before and after change-point.

**Figure 11:**
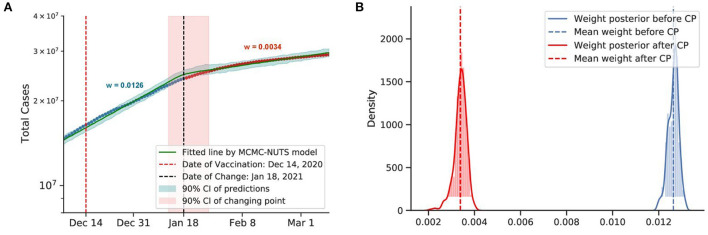
Posterior for the US. The US recorded more than 20 million cases when they start vaccine program on December 14, 2020 (77), US sees the growth rate declined by 73%, from 0.0126 to 0.0034. The change-point point was around January 18, 2021. **(A)** Fitted graph with change-point on January 18, 2021. **(B)** Posterior distribution of weights (transmission rate) before and after change-point.

**Figure 12:**
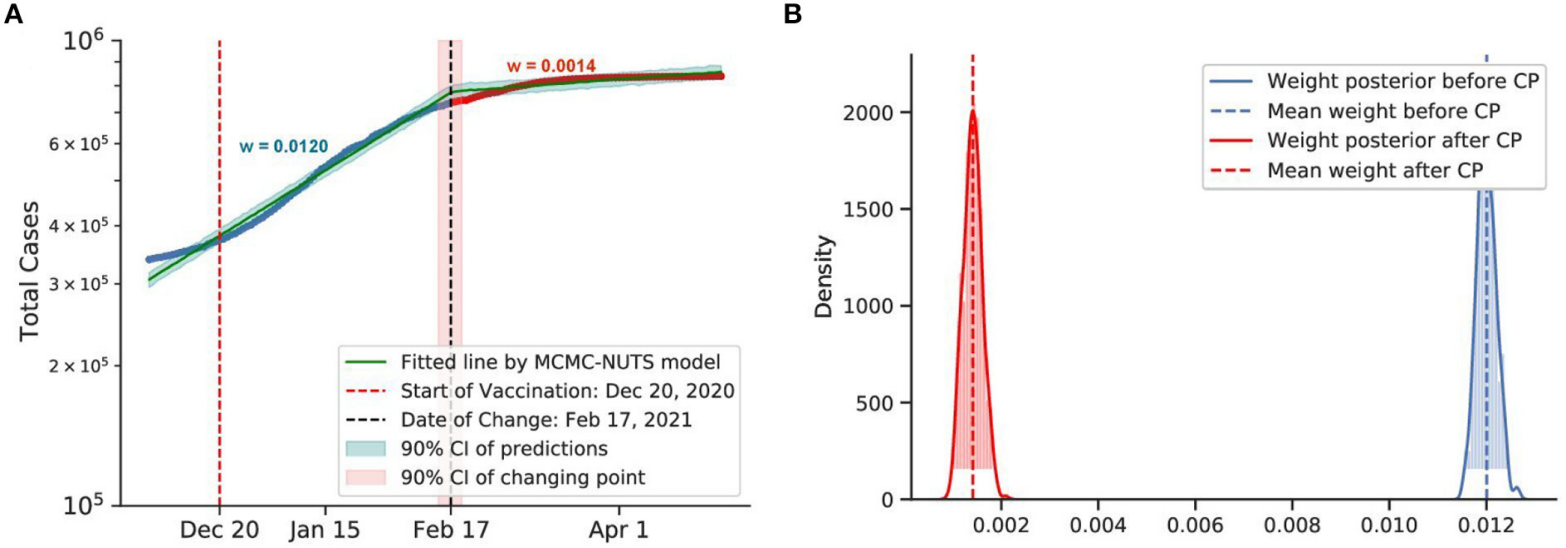
Posterior for Israel. Israel recorded more than 30,000 cases when they started the vaccination program on December 20, 2020 (78). After the vaccination took its affect, the growth rate declined by 88%, from 0.0120 to 0.0014. The change-point point was around February 17, 2021. **(A)** Fitted graph with change-point on February 17, 2021. **(B)** Posterior distribution of weights (transmission rate) before and after change-point.

By using the probabilistic programming model described in the previous section, we detected the amount of time the policy needed to take effect after establishment, the change-point, and policy strength for each case. Since we focused on the initial stage of the pandemic, the time frame we simulated is 3–6 months from the first recorded case, including the date that the policy is enforced and its effect could be seen. In all experiments, results have converged to the values consistent with our priors. The posteriors also fit well with the actual data (Figures 5–12). The summary of policy efficiencies is shown in Table 2. In the next section, we discuss the results in more detail by investigating each policy separately by country.

### 5.4. Discussion of the results of the change-point method

#### 5.4.1. Lockdown

We investigate the lockdown interventions imposed in China and New Zealand. Both countries applied a strict lockdown as their initial strategy to combat the virus spread. The COVID-19 pandemic emerged in China, with the very first case confirmed on December 10, 2019 (70). New Zealand recorded its first case on February 28, 2020 (71).

Both countries experienced a swift reduction in infection after the application of lockdown. With a strong centralized government, China could force a lockdown from January 23, 2020, starting with the epicenter of Wuhan city and Hubei province. The lockdown was overwhelmingly stringent, with a travel ban, a stay-at-home order, and transportation suspension. Other Chinese cities quickly followed suit with similar measures. Our model shows that the policy took its effect around February 8, 2020, with a 98% reduction in the transmission rate.

New Zealand recorded its first case on February 28, 2020. The New Zealand government introduced a four-tier alert level system and imposed a lockdown on most of the country's population and economy from March 25, 2020 (71). The policy seemed to take effect around April 2, 2020, with a 95% reduction in the infection rate.

From these observations, we can conclude that a lockdown is capable of quickly curbing infections. We took the average efficacy of the two mentioned countries, 96% as the efficacy of the lockdown for our further experiments.

#### 5.4.2. Social distancing

We investigated the social distancing imposed in Canada and Singapore. Both countries applied social distancing or soft lockdown mandates in their initial strategy to combat the virus spread. The first COVID-19 case in Canada was confirmed on January 25, 2020 (72). Around March–April, 2020, the Canadian government started to apply several restrictions to maintain social distancing (72).

The first COVID-19 case in Singapore was confirmed on January 2, 2020 (76). The government introduced a soft lockdown (dubbed a circuit-breaker), which included a stay-at-home order and cordon sanitaire^6^. Contact tracing was not extensively utilized until a later stage of the pandemic (73, 79).

Both countries saw a considerable drop in the infection rate. Canada applied the restriction from around March to April, and the policy had an effect on around February 8, 2020, with a 70% reduction in the infection rate. Singapore applied the circuit-breaker on April 7, 2020, and the change-point was determined to be on April 27, 2020, with a 78% reduction in the infection rate.

It is evident that social distancing had a considerable effect on reducing the infection rate. We took the average efficacy of the two mentioned countries, 74%, as the efficacy of social distancing.

#### 5.4.3. Contact tracing and social distancing

Australia and South Korea both utilized a contact tracing strategy coupling with social distancing as their initial strategy to combat the virus spread.

The first COVID-19 case in Australia was confirmed on January 25, 2020. On March 21, 2020, the Australian government imposed social distancing rules, with the closure of “non-essential” services. Swift recruitment of a large contact tracing workforce took place in March 2020 (74).

In South Korea, the first COVID-19 case was confirmed on January 20, 2020 (75). The government raised the alert level to “Serious” on February 25, 2020, announced guidelines to limit trips and outdoor activities and imposed emergency safety measures from basic hygiene rules to self-quarantine and social distancing (76). Health officials implemented extensive movement and contact tracing to identify and inform exposed individuals (76).

Both countries experienced a swift reduction in infection after applying their social distancing coupling with contact tracing. In Australia, the policy seemed to take effect after 10 days (around March 31, 2020), with a 96% reduction in the infection rate. In South Korea, the policy showed effects on March 4, 2020, 8 days after the policy establishment, with an identical reduction in the infection rate.

It is evident that social distancing when coupled with contact tracing can quickly curb the spread of infection. We take the average efficacy of the two mentioned countries, 96%, as the overall efficacy. Thus, contact tracing could push the efficiency of social distancing to the same level as the lockdown.

#### 5.4.4. Effect of mandating masks and hygiene

We could not use the Changing-point model for masks and hygiene because they are hard to separate from other policies. However, we can indirectly represent the transmission rate *via* effective reproduction numbers. The transmission rate is proportional to reproduction number:


$$w=\gamma R_{e}$$

Therefore, we can use the reproduction number ratio.


$$\text{Policy efficiency}=1-\frac{\beta_{2}}{\beta_{1}}=1-\frac{w_{2}}{w_{1}}=1-\frac{R_{e_{2}}}{R_{e_{1}}}$$

We compared the effective reproduction number *R*_*e*_ of Japan before policies were applied with the basic reproduction number *R*_0_ of Sweden from Table 1, which is equal to 2.64.

The reason why we chose Japan lies in its cultural practices, which list the culture of wearing masks, very little physical contact (such as hugging or shaking hands), and not wearing shoes in the house (80). We expect the reproduction number in Japan to be lower even if there is no strict policy applied. The average effective reproduction number *R*_*e*_ for Japan after 6 runs was equal to 1.84. Thus, the efficiency of the hygiene and masks mandates is equal to 1 − 1.84/2.64 = 0.30.

#### 5.4.5. Vaccine

The US and Israel both have a sweeping and widespread vaccination program. The results obtained by our method for the US and Israel are plotted in Figures 11, 12, respectively. The US started the vaccine program on December 14, 2020 (77), while Israel started their campaign on December 20, 2020 (78). These two countries experienced a swift reduction in infection after the vaccine program started. In the US, the policy seemed to show effect around January 18, 2021, with a 73% reduction in the infection rate. In Israel, the policy produced effects on February 18, 2021, with an 88% reduction in transmission rate. According to our results, in both cases, vaccination successfully mitigated the virus spread.

**Figure 13:**
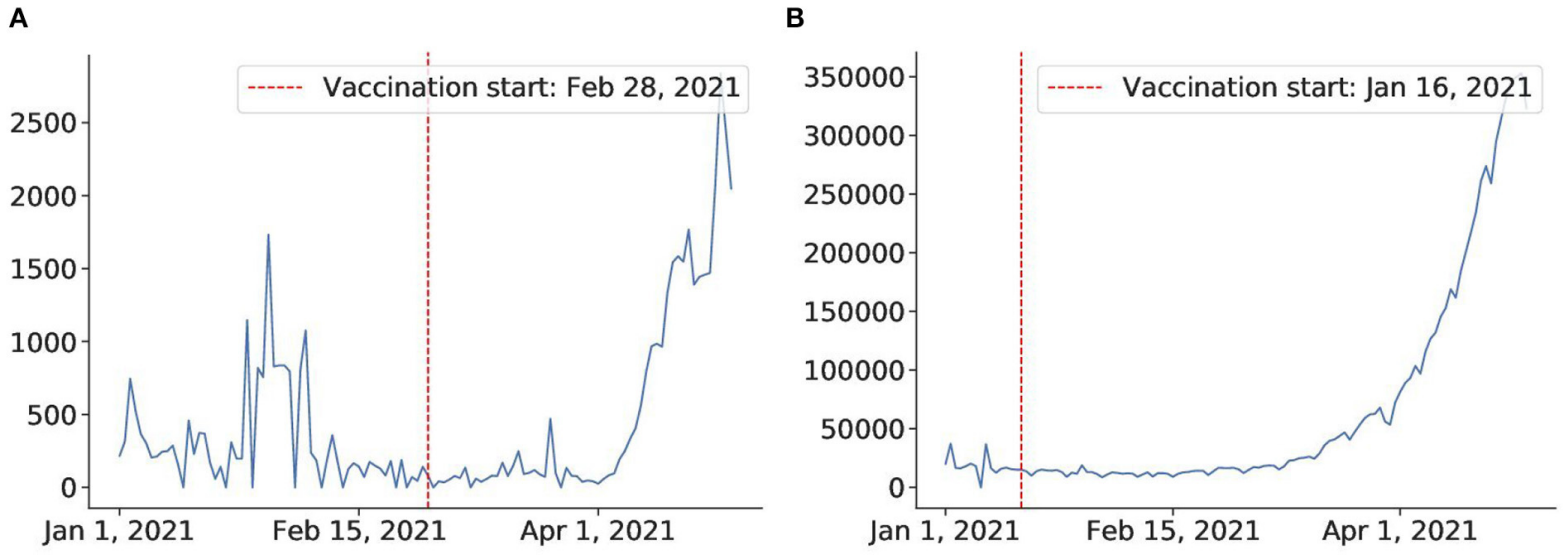
Vaccine fails to mitigate virus in Thailand and India. **(A)** Thailand after vaccination on Feb 28. **(B)** India after vaccination campaign on Jan 16.

However, most countries lack a swift and large-scale vaccination due to different reasons, including delay in vaccine production, financial difficulties, or vaccine hesitancy (81). Thus, in most countries, the fraction of the vaccinated population falls far below the herd immunity threshold according to the current data^7^. The start of vaccination programs can also lead to some incautiousness and fatigue that may have already driven up cases in many countries like India and Thailand. From Figure 13, we can see that after the vaccination campaign started (82, 83) the number of cases increased drastically. It is possible that the reason for such an outcome lies in weakening awareness of coronavirus in the population after the vaccination campaigns start. People may have developed a more relaxed attitude toward restrictions, which consequently may have caused these spikes in confirmed cases. We conclude that large-scale campaigns and accountability of the population in vaccination establishment play a key role in its success.

### 5.5. Discussion

#### 5.5.1. Policy overview

In Section 5, we evaluated the effectiveness of major policies based on the observed statistics. We found that social distancing, lockdown, and contact tracing are all effective in controlling the pandemic, with lockdown having the highest impact on the transmission rate (on average 96% efficacy for China and New Zealand). It was also found that a combination of social distancing with contact tracing was shown to have an effect comparable to the lockdown (also 96% efficacy for South Korea and Singapore). The policy usually takes effect from 8 to 20 days after enforcement.

We also estimated the vaccination campaigns' efficiency. We found that although in countries like Israel and the US, vaccination effectively mitigated the virus spread (on average 81% efficacy for Israel and the US), other countries like Thailand and India failed to bring virus spread under control. Moreover, it seems that vaccination programs were followed by a rapid increase in the confirmed case statistics in such countries. We suppose that reason for such controversial behavior lies in the lack of a large-scale vaccination program, as well as differences in public responsibility awareness.

#### 5.5.2. Limitations

This analysis was based on assumptions, where we ignore the inherent differences between countries and populations. Complex factors such as the acceptance or awareness of the general public could affect the policy's effectiveness. It is evident that some Asian countries tend to perform better in containing the diseases, which we attribute to the collectivist nature and (usually) centralized government. For example, countries with experience with previous epidemics (China and Vietnam with SARS and South Korea with MERS) also tended to perform better thanks to previous experience in handling similar outbreaks.

However, it is too early to conclude that stringent policies like lockdowns are the most successful at mitigating the COVID-19 pandemic since the side effect of applying the policy should also be considered. Considering that the most efficient policies by our estimations may not be the most effective ones in terms of economic cost, we conducted additional experiments to address this issue.

#### 5.5.3. Potential confounding factors

The decrease in caseload is most probably driven by the policy's effect, but it can be due to the shrink of the susceptible population (84). However, since we investigate the policy effect in the initial stage of the pandemic, we assume that the susceptible cases remain relatively stable. The change-point experiments are subjected to the time frame of January to May 2020, and the reported cumulative confirmed cases on May 31, 2020, were about 6 million, which is 0.1% of the global population. Phipps et al. (85) estimated that the number of actual cumulative cases could be 5–6 times larger than the reported number; it does not affect the number of susceptible of the initial stage of the pandemic. The change in transmission rate is principally driven by the policy efficiency, not because of the change of susceptible population.

## 6. Simulation by generative model

Having the virus statistics and policy capacities, we are ready to run our simulation experiments. Our pipeline is flexible enough to handle simulations with different sets of parameters. Since all variables are already inferred, we can use a simple generative model to predict how pandemic plays out in different scenarios. To address the trade-off between public health protection and economic loss, we estimate the cost of the policies and the total loss for given caseloads and death tolls. We tried out multiple policy combinations to figure out what might be the best policy to fight the pandemic in our experimental setting.

### 6.1. Model

To simulate the infection and fatality cases, we used the SEIRD (see Equation 5). We follow the differential equations Equations (1)–(6) and the virus and policy statistic derived in Sections 4, 5. However, the fatality rate will not stay constant as we considered the hospital capacity.

Apart from the parameters related to virus statistics and policy efficiency, we also need the input of the economic effect of the policies as well as the hospital statistics (hospital capacity, percentage of cases requiring hospital admission, and the death rates with and without hospital treatment). Our model gives flexibility to each country to input its own parameters into the model.

### 6.2. Assumption

We illustrate the operation of our model on an imaginary country with a population of 1 million. In addition to the parameters we inferred from previous results, we applied some additional assumptions:

- The hospital capacity is 60 per 100,000 capita (0.06%). Among OECD countries, the number of critical care beds ranges from 3.3 to 33.9 beds per 100,000 capita (86), and the number of hospital beds ranges from 50 to 1,300 beds per 100,000 capita (87). Since countries might adapt normal beds into critical care beds to treat COVID-19 patients amid the health crisis, we use 60 critical care beds per 100,000 capita, which is already double the figure for the most resourceful country (33.9 for Germany).
- 6% of the total cases are required to stay in the intensive care units (ICU).
  Preliminary data on a subset of 7,162 COVID-19 patients age 19 and older with known health history in the US, from November 12, 2020, to March 28, 2020, found that 6% requires ICU treatment^8^.
- ICU-required cases will die without ICU treatment. With treatment, the death rate for cases admitted to ICU is 60%.
  Data from Washington, Seattle, and California suggests mortality rates reported in patients with severe COVID-19 in the ICU range from 50 to 65% (88).

We also estimate the economic and human capital cost for each policy:

- Lockdown: 10% of GDP per year
- Distancing: 5% of GDP per year
- Hygiene and masks: $2 per day per capita.
- Infection: $300 per infection per day (until recovered).
- Contact-tracing: $6,400 per new case.
- Death: $7 million per death.

These estimations are reasonably set based on the following facts:

- Research suggests that global output shrinks by about 33% at the peak of a lockdown, with an annual impact of over 9% of the annual GDP (3).
- The value of one human life is estimated to be A$5.0 M ($3.48 M) in Australia in 2020 (89) and $10 M in America in 2017 (90).
- In South Korea, the treatment's average daily cost for a mild patient is 180,000–260,000 ($158–$229), and for severe patients is 650,000 ($572) (91).
- The costs of a contact tracing policy include the administrative (monetary) cost and the total quarantine days of the second-generation contacts. The standard contact tracing policy, where all close contacts are requested to quarantine for 14 days from the day of exposure, is estimated to cost 62.1 quarantine days and $189 per index case (92). We assume contact tracing is done on every confirmed case, and each quarantine day costs $100 (South Korea's government quarantine facility costs 100,000–150,000₩ ($88–$130) per day)^9^. The total costs can be estimated as about $6,400 per case ($6,210 for 62.1 quarantine days and $189 for administrative cost).
- Price of a mask in South Korea is normally set around $2 and $1.2 under the government rationing scheme (93).

### 6.3. Lockdown only delays the virus spread

**Figure 14:**
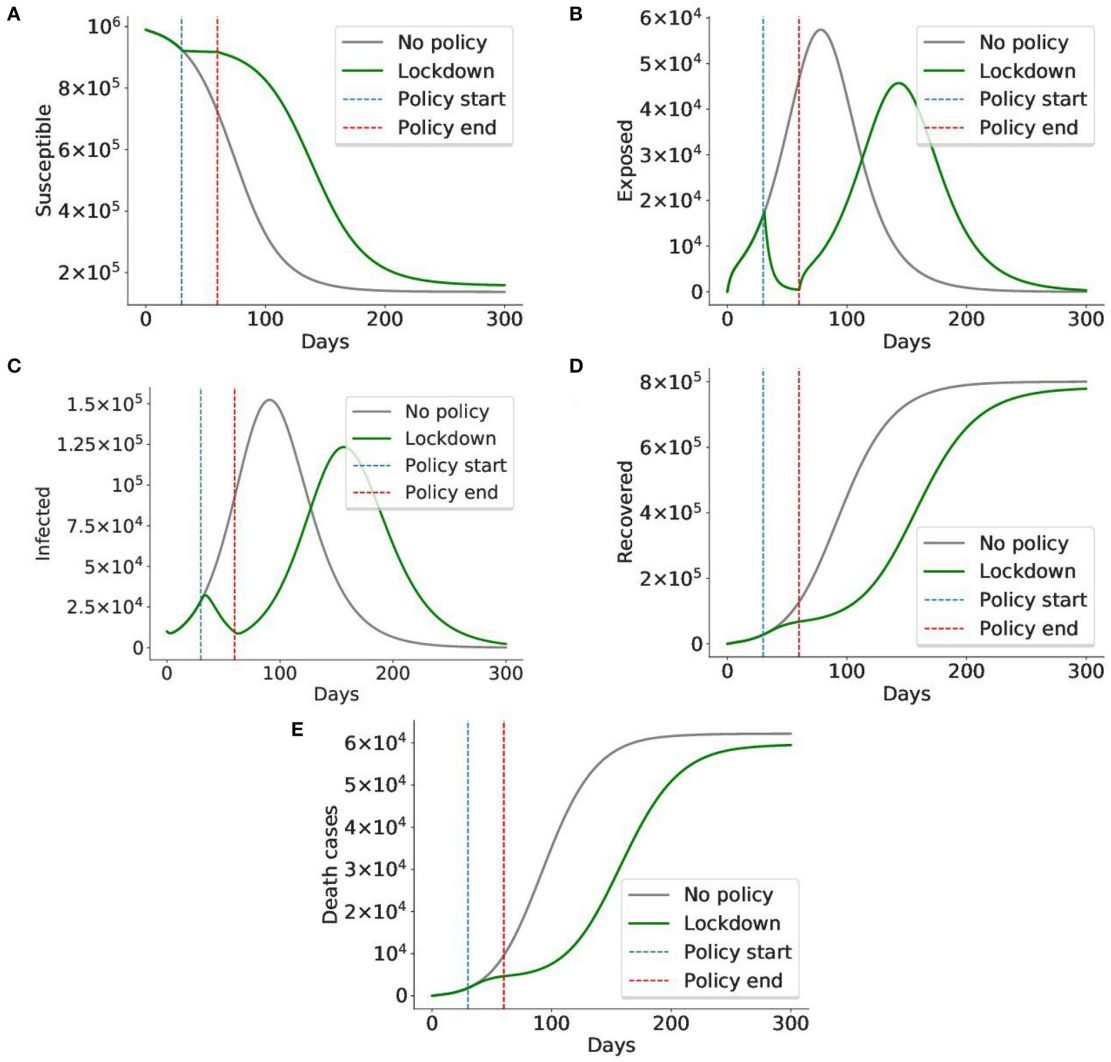
A lockdown delays the virus spread, but cannot prevent it. In this simulation, the lockdown policy was imposed from day 30 to day 60 (total 1 month). The gray graph represents the baseline situation with no policy applied, the green graph indicates the case when a lockdown is applied. The blue and red lines mark the start and end of the lockdown, respectively. We can see that although lockdown sharply decreases the number of exposed and infected cases, it cannot prevent the virus from spreading after the lockdown is lifted on day 60. The number of exposed and infected cases rises again. Given the cost of the lockdown, it is impossible to maintain it for long periods of time, making it less preferable to less costly policies. Thus, the conclusion is in alignment with those of prior works (42, 46). **(A)** Susceptible cases, **(B)** Exposed cases, **(C)** Infected cases, **(D)** Recovered cases, **(E)** Death cases.

We ran the model without any policy and with the lockdown applied from day 30 to 60. As you can see from Figure 14, applying lockdown for 1 month simply postpones the virus spread. Another problem is that it significantly hits the country's economy, so it cannot be applied for a long time. Thus, even though lockdown is estimated to have the highest efficiency of 0.96, it might not be the best policy to apply. So further experiments are required to identify how, when, and for how long the policies should be applied.

### 6.4. Best initial response: Social distancing with contact tracing

Finally, we want to devise the best initial response to the virus. Using the inferred statistics, we conducted experiments on the policies and performed simulations to develop the optimal policy with minimal loss (both economic loss and life loss). We designed an imaginary country with a population of 1 million and a GDP of $30,000 per capita. The country had a population of 1 million, and the simulation spanned three months. We assume that policy-makers revised the policy every month, and a policy is applied exhaustively, partially (50% efficacy), or not applied at all. Policies could be applied together. The goal was to minimize the cost.

#### 6.4.1. Results

**Figure 15:**
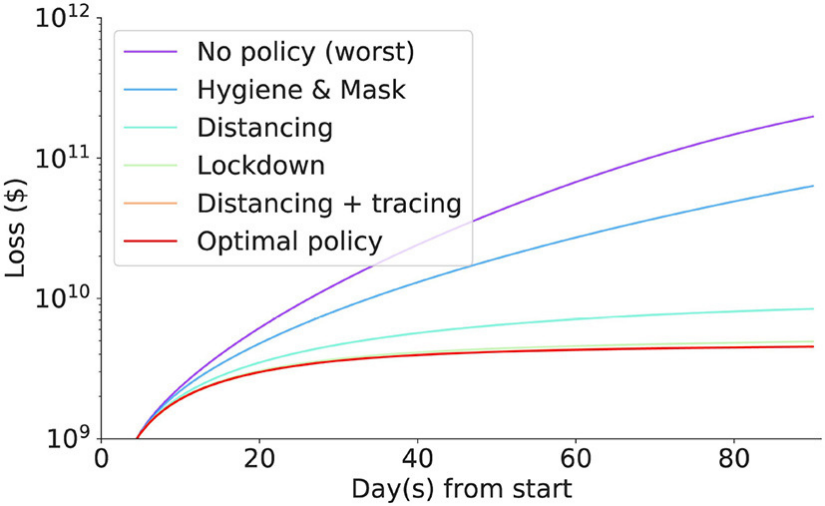
The (daily) accumulated loss incurred in each intervention. Social distancing coupled with contact tracing incurred the least loss, followed by lockdown, social distancing, masks and hygiene mandates, and no policy incurring the biggest loss.

Then results after three months for some important policy combinations are shown in Table 3. The full loss trajectories of important policies are shown in Figure 15.

**Table 3:** Loss regarding some applied policies, full results are available in our repository.

**Policy combination**	**Total cases**	**Total deaths**	**Total loss (billion $)**
Optimal policy*	10,734	577	4.526
Contact tracing and distancing	11,003	591	4.569
Lockdown	11,003	591	4.933
Social distancing	22,478	1,138	8.437
Mask and hygiene mandate	201,929	8,941	63.400
No policy	592,136	28,018	197.927

*Optimal policy: Contact tracing and distancing for three months with additional hygiene and mask mandate for the first month.

The best policy identified so far is contact tracing with social distancing, with a loss of around 2 billion dollars. Without intervention, the loss in the imaginary nation is $197.9 billion. Scaling up to the US population, the simulated economic loss reaches $65 trillion which is nearly equivalent to the World GDP of $85 trillion, implying that intervention must be enforced in the initial stage of the pandemic.

Generally, social distancing coupling with contact tracing incurs less loss than lockdown or social distancing. They are all strong interventions compared to masks and hygiene mandates. However, they all significantly save a tremendous loss compared to doing nothing or only doing the mask and hygiene mandates. The human cost for mild intervention seems to be significantly lower than the economic cost of the strong intervention. Nonetheless, the masks and hygiene mandates still halved the loss that we suffer when we do nothing.

Contact tracing coupled with social distancing reduces the economic and human capital loss by 98% compared to doing nothing. Although as efficient as lockdown (Section 5), the economic and human capital costs are at least 8% less in a 3 month period. The *optimal* policy in our setting is contact tracing and social distancing for three months with additional hygiene and masks mandates for the first month. Hygiene and masks mandates play some role in minimizing the loss, albeit the improvement is marginal.

Therefore, we can conclude that quarantine and contact tracing are the most efficient policies in our setting. Indeed, we can see that countries that enjoyed the initial success in controlling the virus cases, e.g., South Korea, Vietnam, or Australia applied social distancing and contact tracing as their primary policies.

### 6.5. Limitations and implications

Due to the challenge of separating the policy effects, our study has some limitations within which our findings need to be interpreted carefully. First, we have investigated major policies applied in relatively wealthy countries. For economic loss estimations, we utilized policies' costs reported from various developed countries using different sources. Depending on the parameters adjusted for a particular country, the results might be different. Second, the estimated efficiency of each policy in Section 5 is measured on the most successful cases. Although it provides a meaningful upper bound for each policy's efficiency, we cannot simply assume that every country can achieve this maximum efficiency. Therefore, the “best” initial response in Section 6.4 should be understood under the context that every policy can be feasibly applied to its fullest extent. Third, future work can utilize our model and extend it by considering the confounding effects of other interventions or changes in the susceptible population size. Such a model can be used to estimate the efficiencies of the policies applied in the later stages of the pandemic.

Given the unique socioeconomic state, each country has its own feasible stringency and the price tag for each measure. Many factors contribute to this variability, such as public acceptance, political climate, and government priority. To cope with that, we provide a flexible end-to-end pipeline, which can be tailored to each country's specific needs. Decision-makers can adjust the corresponding parameters or apply their country's cost estimation to adapt to their own situation. They can also exclude infeasible policy settings in their country when running the simulation. The resilience to control model's parameters allows countries to see how the pandemic will play out under different scenarios and build their own strategies based on the model's output.

## 7. Conclusion

Recent research on COVID-19 propagation analysis has provided a deeper understanding of the transmission processes occurring during the past 1.5 years. Epidemiological models point out the key factors that affect the spread of the virus, including the basic reproduction number, virus incubation period, and daily infection number. In the present study, we have moved one step further to gauge the efficacy of the early-stage policy to respond to the pandemic, with economic factors related to the policy itself and its benefits of slowing down the virus. Detailed analysis from 10 countries suggests that social distancing, coupled with contact tracing, is the most efficient policy among major initial interventions. From the data of Asian countries, we derive meaningful results that close contact tracing could provide protection to citizens from the pandemic comparable to lockdowns, without inducing as much cost. Going one step further, we carefully designed a simulated country and gauged the efficacy of each policy combination. Our testbed allows end-users to control various parameters suitable for their country's situation. Through the process of overcoming COVID-19, we are gaining a clearer understanding of the trade-off between virus prevention and economic loss. As we have seen in many countries, it is crucial to identify each policy's efficiencies and costs and to estimate the best time and intensity to impose them before it is too late. We hope that our research will assist every nation in responding to possible future pandemics.

## Data availability statement

The original contributions presented in the study are included in the article/supplementary material, further inquiries can be directed to the corresponding author.

## Author contributions

AZ and T-DM: conceptualization and data acquisition. All authors: methodology, writing, review, editing, final approval, and accountability for accuracy and integrity. All authors contributed to the article and approved the submitted version.

## Funding

This work was supported by the Institute for Basic Science (IBS-R029-C2, IBS-R029-Y4) and the Institute of Information and communications Technology Planning and Evaluation (IITP) grant funded by the Korea government (MSIT) [No.2019-0-01842, Artificial Intelligence Graduate School Program (GIST)].

## Conflict of interest

T-DM was employed by the company Samsung Electronics, but this work was done when he was an undergraduate student at KAIST/IBS Data Science Group.

The remaining authors declare that the research was conducted in the absence of any commercial or financial relationships that could be construed as a potential conflict of interest.

## Publisher's note

All claims expressed in this article are solely those of the authors and do not necessarily represent those of their affiliated organizations, or those of the publisher, the editors and the reviewers. Any product that may be evaluated in this article, or claim that may be made by its manufacturer, is not guaranteed or endorsed by the publisher.

## Footnotes

^1^ https://www.kaggle.com/imdevskp/corona-virus-report?select=full_grouped.csv

^2^ https://coronavirus.jhu.edu/map.html

^3^ https://www.worldometers.info/coronavirus/

^4^ https://git.io/Jvoxz

^5^This work does not involve any human participant, thus not subject to the IRB approval.

^6^A cordon sanitaire is the restriction of movement of people into or out of a defined geographic area, such as a community, region, or country.

^7^ https://ourworldindata.org/grapher/share-people-fully-vaccinated/~covid

^8^ https://www.tfah.org/wp-content/uploads/2020/04/COVIDunderlyingconditions040320.pdf

^9^ https://kr.usembassy.gov/022420-covid-19-information/

## References

[1] Dong E Du H Gardner L. An interactive web-based dashboard to track COVID-19 in real time. Lancet Infect Dis. (2020) 20:533–4. 10.1016/S1473-3099(20)30120-1 32087114 PMC7159018

[2] Wheelock DC. Comparing the COVID-19 recession with the great depression. Econom Synopses. (2020) 39. 10.20955/es.2020.39

[3] Mandel A Veetil V. The economic cost of COVID lockdowns: an out-of-equilibrium analysis. Econ Disast Clim Change. (2020) 4:431–51. 10.1007/s41885-020-00066-z 32838118 PMC7304379

[4] Inoue H Todo Y. Propagation of the Economic Impact of Lockdowns Through Supply Chains. VOX, CEPR Policy Portal (2020) 10.1371/journal.pone.0255031

[5] Pons O. Estimations and Tests in Change-Point Models. Singapore: World Scientific (2018). 10.1142/10757

[6] Aminikhanghahi S Cook DJ. A survey of methods for time series change point detection. Knowl Inform Syst. (2017) 51:339–67. 10.1007/s10115-016-0987-z 28603327 PMC5464762

[7] Rajakumar K Weisse M. Centennial year of Ronald Ross' epic discovery of malaria transmission: an essay and tribute. Southern Med J. (1999) 92:567–71. 10.1097/00007611-199906000-00004 10372849

[8] Ross R. Some quantitative studies in epidemiology. Nature. (1911) 87:466–7. 10.1038/087466a0

[9] Brauer F. In: Brauer F, van den Driessche P, Wu J, editors. Compartmental Models in Epidemiology. Berlin; Heidelberg: Springer Berlin Heidelberg (2008). p. 19–79. 10.1007/978-3-540-78911-6_2

[10] Li MY Muldowney JS. Global stability for the SEIR model in epidemiology. Math Biosci. (1995) 125:155–64. 10.1016/0025-5564(95)92756-5 7881192

[11] Annas S Isbar Pratama M Rifandi M Sanusi W Side S. Stability analysis and numerical simulation of SEIR model for pandemic COVID-19 spread in Indonesia. Chaos Solitons Fract. (2020) 139:110072. 10.1016/j.chaos.2020.110072 32834616 PMC7345386

[12] Pandey G Chaudhary P Gupta R Pal S. SEIR and regression model based COVID-19 outbreak predictions in India. arXiv preprint arXiv:200400958. (2020). 10.2196/preprints.19406

[13] Yang Z Zeng Z Wang K Wong SS Liang W Zanin M. Modified SEIR and AI prediction of the epidemics trend of COVID-19 in China under public health interventions. J Thorac Dis. (2020) 12:165. 10.21037/jtd.2020.02.64 32274081 PMC7139011

[14] Viguerie A Lorenzo G Auricchio F Baroli D Hughes TJ Patton A. Simulating the spread of COVID-19 *via* a spatially-resolved susceptible-exposed-infected-recovered-deceased (SEIRD) model with heterogeneous diffusion. Appl Math Lett. (2021) 111:106617. 10.1016/j.aml.2020.106617 32834475 PMC7361091

[15] Piccolomini EL Zama F. Monitoring Italian COVID-19 spread by a forced SEIRD model. PLoS ONE. (2020) 15:e0237417. 10.1371/journal.pone.0237417 32760133 PMC7410324

[16] Metropolis N Rosenbluth AW Rosenbluth MN Teller AH Teller E. Equation of state calculations by fast computing machines. J Chem Phys. (1953) 21:1087–92. 10.1063/1.1699114 41342507

[17] Hastings WK. Monte Carlo sampling methods using Markov chains and their applications. Biometrika. (1970) 57:97–109. 10.1093/biomet/57.1.97

[18] Eraker B. MCMC analysis of diffusion models with application to finance. J Bus Econ Stat. (2001) 19:177–91. 10.1198/073500101316970403

[19] Johannes M Polson N. MCMC methods for continuous-time financial econometrics. In:Ait-Sahalia Y Hasen LP, editors. Handbook of Financial Econometrics: Applications. Amsterdam: Elsevier (2010). p. 1–72. 10.1016/B978-0-444-53548-1.50003-9

[20] Farhang-Boroujeny B Zhu H Shi Z. Markov chain Monte Carlo algorithms for CDMA and MIMO communication systems. IEEE Trans Signal Process. (2006) 54:1896–909. 10.1109/TSP.2006.872539

[21] Al-Qaq WA Devetsikiotis M Townsend JK. Stochastic gradient optimization of importance sampling for the efficient simulation of digital communication systems. IEEE Trans Commun. (1995) 43:2975–85. 10.1109/26.477500

[22] Gupta A Rawlings JB. Comparison of parameter estimation methods in stochastic chemical kinetic models: examples in systems biology. AIChE J. (2014) 60:1253–68. 10.1002/aic.14409 27429455 PMC4946376

[23] Gill J. Is partial-dimension convergence a problem for inferences from MCMC algorithms? Polit Anal. (2008) 16:153–78. 10.1093/pan/mpm019

[24] Wells MT Casella G Robert CP. Generalized accept-reject sampling schemes. In:DasGupta A, editor. A Festschrift for Herman Rubin. Institute of Mathematical Statistics (2004). p. 342–7. 10.1214/lnms/1196285403

[25] Hamra G MacLehose R Richardson D. Markov chain Monte Carlo: an introduction for epidemiologists. Int J Epidemiol. (2013) 42:627–34. 10.1093/ije/dyt043 23569196 PMC3619958

[26] Lawson AB. MCMC methods for putative pollution source problems in environmental epidemiology. Stat Med. (1995) 14:2473–85. 10.1002/sim.4780142115 8711282

[27] Ramkissoon J,. Detecting Changes in COVID-19 Cases with Bayesian Models. (2020). Available online at: https://bit.ly/2Qd7GCN (accessed October 27, 2022).

[28] Cauchemez S Carrat F Viboud C Valleron A Boelle P. A Bayesian MCMC approach to study transmission of influenza: application to household longitudinal data. Stat Med. (2004) 23:3469–87. 10.1002/sim.1912 15505892

[29] Lekone PE Finkenstädt BF. Statistical inference in a stochastic epidemic SEIR model with control intervention: Ebola as a case study. Biometrics. (2006) 62:1170–7. 10.1111/j.1541-0420.2006.00609.x 17156292

[30] Ndanguza D Tchuenche J Haario H. Statistical data analysis of the 1995 Ebola outbreak in the Democratic Republic of Congo. Afrika Mat. (2013) 24:55–68. 10.1007/s13370-011-0039-59988161

[31] Zhou T Ji Y. Semiparametric Bayesian inference for the transmission dynamics of COVID-19 with a state-space model. Contemp Clin Trials. (2020) 97:106146. 10.1016/j.cct.2020.106146 32947047 PMC7491370

[32] Brooks S. Markov chain Monte Carlo method and its application. J R Stat Soc Ser D. (1998) 47:69–100. 10.1111/1467-9884.00117

[33] Mbuvha R Marwala T. Bayesian inference of COVID-19 spreading rates in South Africa. PLoS ONE. (2020) 15:e0237126. 10.1371/journal.pone.0237126 32756608 PMC7406053

[34] Dehning J Zierenberg J Spitzner FP Wibral M Neto JP Wilczek M. Inferring COVID-19 spreading rates and potential change points for case number forecasts. Science. (2020) 369:eabb9789. 10.1126/science.abb9789 32414780 PMC7239331

[35] Kavaliunas A Ocaya P Mumper J Lindfeldt I Kyhlstedt M. Swedish policy analysis for COVID-19. Health Policy Technol. (2020) 9:598–612. 10.1016/j.hlpt.2020.08.009 32904437 PMC7455549

[36] Debnath R Bardhan R. India nudges to contain COVID-19 pandemic: a reactive public policy analysis using machine-learning based topic modelling. PLoS ONE. (2020) 15:e0238972. 10.1371/journal.pone.0238972 32915899 PMC7485898

[37] Naumann E Möhring K Reifenscheid M Wenz A Rettig T Lehrer R. COVID-19 policies in Germany and their social, political, and psychological consequences. Eur Policy Anal. (2020) 6:191–202. 10.1002/epa2.1091 34616900 PMC7537296

[38] Chinazzi M Davis JT Ajelli M Gioannini C Litvinova M Merler S. The effect of travel restrictions on the spread of the 2019 novel coronavirus (COVID-19) outbreak. Science. (2020) 368:395–400. 10.1126/science.aba9757 32144116 PMC7164386

[39] Arenas A Cota W Gomez-Gardenes J Gómez S Granell C Matamalas JT. Derivation of the effective reproduction number R for COVID-19 in relation to mobility restrictions and confinement. medRxiv. (2020). 10.1101/2020.04.06.20054320

[40] Teslya A Pham TM Godijk NG Kretzschmar ME Bootsma MC Rozhnova G. Impact of self-imposed prevention measures and short-term government-imposed social distancing on mitigating and delaying a COVID-19 epidemic: a modelling study. PLoS Med. (2020) 17:e1003166. 10.1371/journal.pmed.1003166 32692736 PMC7373263

[41] Iwata K Doi A Miyakoshi C. Was school closure effective in mitigating coronavirus disease 2019 (COVID-19)? Time series analysis using Bayesian inference. Int J Infect Dis. (2020) 99:57–61. 10.1016/j.ijid.2020.07.052 32745628 PMC7836901

[42] Sharov KS. Creating and applying SIR modified compartmental model for calculation of COVID-19 lockdown efficiency. Chaos Solitons Fract. (2020) 141:110295. 10.1016/j.chaos.2020.110295 32994671 PMC7513696

[43] Hale T Petherick A Phillips T Webster S. Variation in Government Responses to COVID-19. Blavatnik school of government working paper, Vol. 31. University of Oxford (2020).

[44] Jinjarak Y Ahmed R Nair-Desai S Xin W Aizenman J. Accounting for global COVID-19 diffusion patterns, January-April 2020. Econ Disast Clim Change. (2020) 4:515–59. 10.1007/s41885-020-00071-2 32901228 PMC7471593

[45] Flaxman S Mishra S Gandy A Unwin HJT Mellan TA Coupland H. Estimating the effects of non-pharmaceutical interventions on COVID-19 in Europe. Nature. (2020) 584:257–61. 10.1038/s41586-020-2405-7 32512579

[46] Haug N Geyrhofer L Londei A Dervic E Desvars-Larrive A Loreto V. Ranking the effectiveness of worldwide COVID-19 government interventions. Nat Hum Behav. (2020) 4:1303–12. 10.1038/s41562-020-01009-0 33199859

[47] Brauner JM Mindermann S Sharma M Johnston D Salvatier J Gavenčiak T. Inferring the effectiveness of government interventions against COVID-19. Science. (2021) 371:6531. 10.1126/science.abd9338 33323424 PMC7877495

[48] Singh S Shaikh M Hauck K Miraldo M. Impacts of introducing and lifting nonpharmaceutical interventions on COVID-19 daily growth rate and compliance in the United States. Proc Natl Acad Sci. USA. (2020) 118:e2021359118. 10.1073/pnas.2021359118 33658331 PMC8000285

[49] Maital S Barzani E. The Global Economic Impact of COVID-19: A Summary of Research. Samuel Neaman Institute for National Policy Research (2020). p. 1–12.

[50] McKibbin W Fernando R. The Economic Impact of COVID-19. Economics in the Time of COVID-19. CEPR Press (2020). p. 45.

[51] McKibbin W Fernando R. The Global Macroeconomic Impacts of COVID-19: Seven Scenarios. Centre for Applied Macroeconomic Analysis. Asian Economic Papers (2020). p. 1–55. 10.2139/ssrn.3547729

[52] Manski CF Molinari F. Estimating the COVID-19 infection rate: anatomy of an inference problem. J Econ. (2021) 220:181–92. 10.1016/j.jeconom.2020.04.041 32377030 PMC7200382

[53] Korolev I. Identification and estimation of the SEIRD epidemic model for COVID-19. J Econ. (2021) 220:63–85. 10.1016/j.jeconom.2020.07.038 32836680 PMC7392128

[54] Hortaçsu A Liu J Schwieg T. Estimating the fraction of unreported infections in epidemics with a known epicenter: an application to COVID-19. J Econ. (2021) 220:106–29. 10.1016/j.jeconom.2020.07.047 32921876 PMC7476454

[55] Bingham E Chen JP Jankowiak M Obermeyer F Pradhan N Karaletsos T. Pyro: deep universal probabilistic programming. J Mach Learn Res. (2018) 20:973–8. 10.5555/3322706.3322734

[56] Pyro. Epidemiology. (2021). Available online at: https://bit.ly/3fiv2kq (accessed October 27, 2022).

[57] Andrieu C De Freitas N Doucet A Jordan MI. An introduction to MCMC for machine learning. Mach Learn. (2003) 50:5–43. 10.1023/A:1020281327116

[58] Homan MD Gelman A. The No-U-Turn sampler: adaptively setting path lengths in Hamiltonian Monte Carlo. J Mach Learn Res. (2014) 15:1593–623. 10.48550/arXiv.1111.4246

[59] Betancourt MJ Girolami M. Hamiltonian Monte Carlo for hierarchical models. Current Trends in Bayesian Methodology with Applications. (2015) 79:2–4.

[60] Neal RM. MCMC using Hamiltonian dynamics. Handbook of Markov Chain Monte Carlo. (2012) 2:2. 10.1201/b10905-6

[61] Betancourt M. Diagnosing suboptimal cotangent disintegrations in Hamiltonian Monte Carlo. arXiv [Preprint]. arXiv:160400695. (2016). 10.48550/arXiv.1604.00695

[62] Fottrell Q. Sweden Embraced Herd Immunity, While the U.K. Abandoned the Idea-So Why Do They Both Have High COVID-19 Fatality Rates? (2020). Available online at: https://on.mktw.net/34dOR66 (accessed October 27, 2022).

[63] WHO. Coronavirus Disease (COVID-19). World Health Organization (2020). Available online at: https://bit.ly/34ic0Ey (accessed October 27, 2022).

[64] Parag KV Thompson RN Donnelly CA. Are epidemic growth rates more informative than reproduction numbers? J R Stat Soc Ser A. (2022) 1–11. 10.1111/rssa.12867 35942192 PMC9347870

[65] Abraham J Turville C Dowling K Florentine S. Does climate play any rin COVID-19 spreading?—An Australian perspective. Int J Environ Res Public Health. (2021) 18:9086. 10.3390/ijerph18179086 34501673 PMC8431748

[66] Gebski V Ellingson K Edwards J Jernigan J Kleinbaum D. Modelling interrupted time series to evaluate prevention and control of infection in healthcare. Epidemiol Infect. (2012) 140:2131–41. 10.1017/S0950268812000179 22335933 PMC9152341

[67] Briegel T Tresp V. Robust neural network regression for offline and online learning. In: Advances in Neural Information Processing Systems. Denver (1999). p. 407–13.

[68] Kim JH Marks F Clemens JD. Looking beyond COVID-19 vaccine phase 3 trials. Nat Med. (2021) 27:205–11. 10.1038/s41591-021-01230-y 33469205

[69] Jones I Roy P. Sputnik V COVID-19 vaccine candidate appears safe and effective. Lancet. (2021) 397:642–3. 10.1016/S0140-6736(21)00191-4 33545098 PMC7906719

[70] Crossley G. Wuhan Lockdown 'Unprecedented', Shows Commitment to Contain Virus: WHO Representative in China. Reuters (2020). Available online at: https://tinyurl.com/26et3nd3 (accessed October 27, 2022).

[71] Jong Ed. '*Kiwis*- *Go Home': New Zealand To Go Into Month-Long Lockdown to Fight Coronavirus*. The Guardian (2021). Available online at: https://tinyurl.com/3nvrd8e6 (accessed October 27, 2022).

[72] Urrutia D Manetti E Williamson M Lequy E. Overview of Canada's answer to the COVID-19 pandemic's first wave (January-April 2020). Int J Environ Res Public Health. (2021) 18:7131. 10.3390/ijerph18137131 34281075 PMC8297373

[73] SSO. COVID-19 (Temporary Measures) (Control Order) Regulations 2020. Singapore Statutes Online (2020). Available online at: https://tinyurl.com/3k34wttr (accessed October 27, 2022).

[74] Guardian. PM Announces Pubs, Clubs and Cinemas to Close, Schools Stay Open in Stage One Measures - As It Happened. The Guardian (2020). Available online at: https://tinyurl.com/yb7v8pkm (accessed October 27, 2022).

[75] MOHW. The First Imported Case of the Novel Coronavirus (2019-nCoV) in Korea: Press Release: News room. The Ministry of Health and Welfare (MOHW) (2020). Available online at: https://tinyurl.com/38r74y8n (accessed October 27, 2022).

[76] Lee D Lee J. Testing on the move: South Korea's rapid response to the COVID-19 pandemic. Transp Res Interdiscip Perspect. (2020) 5:100111. 10.1016/j.trip.2020.100111 34171015 PMC7172645

[77] Painter EM Ussery EN Patel A Hughes MM Zell ER Moulia DL. Demographic characteristics of persons vaccinated during the first month of the COVID-19 vaccination program-United States, December 14, 2020-January 14, 2021. Morbid Mortal Wkly Rep. (2021) 70:174. 10.15585/mmwr.mm7005e1 33539333 PMC7861480

[78] Rinott E Youngster I Lewis YE. Reduction in COVID-19 patients requiring mechanical ventilation following implementation of a national COVID-19 vaccination program-Israel, December 2020-February 2021. Morbid Mortal Wkly Rep. (2021) 70:326. 10.15585/mmwr.mm7009e3 33661863 PMC7948930

[79] MCI. Ending Circuit Breaker: Phased Approach to Resuming Activities Safely. MCI - GovSG (2020). Available online at: https://tinyurl.com/bdfawabj (accessed October 27, 2022).

[80] Kopp R. Does Japan's Culture Explain Its Low COVID-19 Numbers? The Japan Times (2020). Available online at: https://bit.ly/3wuua22 (accessed October 27, 2022).

[81] Wouters OJ Shadlen KC Salcher-Konrad M Pollard AJ Larson HJ Teerawattananon Y. Challenges in ensuring global access to COVID-19 vaccines: production, affordability, allocation, and deployment. Lancet. (2021) 397:1023–34. 10.1016/S0140-6736(21)00306-8 33587887 PMC7906643

[82] Wongcha-um P. Thailand Starts COVID-19 Vaccination Campaign. Reuter News (2021). Available online at: https://reut.rs/3umlNUG (accessed March 29, 2021).

[83] WHO. COVID-19 Vaccination, Jammu and Kashmir, Cowin App. World Health Organization (2022). Available online at: https://tinyurl.com/2p8ur43s (accessed October 27, 2022).

[84] Korolev I. On Reduced Form Estimation of the Effect of Anti-Contagion Policies on the COVID-19 Pandemic. Working Paper, Binghamton University (2020). 10.2139/ssrn.3659623

[85] Phipps SJ Grafton RQ Kompas T. Robust estimates of the true (population) infection rate for COVID-19: a backcasting approach. R Soc Open Sci. (2020) 7:200909. 10.1098/rsos.200909 33391791 PMC7735365

[86] OECD. Beyond Containment: Health Systems Responses to COVID-19 in the OECD. OECD (2020).

[87] OECD. Hospital Beds. (2018) Available online at: https://www.oecd-ilibrary.org/content/data/0191328e-en

[88] Oliveira E Parikh A Lopez-Ruiz A Carrilo M Goldberg J Cearras M. ICU outcomes and survival in patients with severe COVID-19 in the largest health care system in central Florida. PLoS ONE. (2021) 16:e0249038. 10.1101/2020.08.25.20181909 33765049 PMC7993561

[89] OBPR. Best Practice Regulation Guidance Note: Value of Statistical Life. The Office of Best Practice Regulation (2020). Available online at: https://bit.ly/3unsGoO (accessed October 27, 2022).

[90] Kniesner TJ Viscusi WK. The value of a statistical life. Vanderbilt Law Res Pap. (2019) 19:1–45. 10.1093/acrefore/9780190625979.013.138

[91] Lee H,. Korea's COVID-19 Treatment Costs far Lower Than US. (2020). Available online at: https://bit.ly/3oOPKvB (accessed October 27, 2022).

[92] Perrault A Charpignon M Gruber J Tambe M Majumder M. Designing efficient contact tracing through risk-based quarantining. National Bureau of Economic Research. No 28135. (2020). 10.3386/w28135

[93] Glazer A. Price controls don't work - but mask rationing is the exception that proves the rule (2021) Available online at: https://bit.ly/3hYHzLA (accessed October 27, 2022).

